# A food‐based approach could improve dietary adequacy for 12–23‐month‐old Eastern Ugandan children

**DOI:** 10.1111/mcn.13311

**Published:** 2022-01-03

**Authors:** Njeri C. Kimere, Joweria Nambooze, Haeun Lim, Andrea L.S. Bulungu, Kate Wellard, Elaine. L. Ferguson

**Affiliations:** ^1^ Department of Population Health London School of Hygiene and Tropical Medicine London UK; ^2^ Department of Food Systems and Nutrition Africa Innovations Institute Kampala Uganda; ^3^ Livelihoods and Institutions Department, Natural Resources Institute University of Greenwich, Medway Campus Chatham UK

**Keywords:** dietary adequacy, food choice, food‐based recommendations, nutrients, nutrition, Uganda, young children

## Abstract

Little is known about dietary adequacy, for young Ugandan children, or context‐specific food choices to improve it. This study estimated the percentage of breastfed 12–23‐month‐old rural Eastern Ugandan children (*n* = 114) at risk of inadequate intakes of 12 nutrients; and identified realistic food choices for improving it. In this cross‐sectional survey, dietary (weighed food records), anthropometric and socioeconomic data were collected. The percentages of children at risk of inadequate nutrient intakes were estimated, assuming 541 g/day of breast milk was consumed. The median nutrient densities of their complementary feeding diets were also compared with desired levels. Linear programming analyses were used to identify ‘problem nutrients’ (where requirements will be difficult to meet given dietary practices) and model food choices to improve dietary adequacy. Overall, 21.2% of children were stunted and 3.8% were wasted. A high percentage (>45%) of children were at risk of inadequate intakes, for nine of the 12 nutrients assessed, and dietary nutrient densities were below desired levels for seven of the 12 nutrients. Iron, calcium, thiamine and niacin were ‘problem nutrients’. Through careful selection of foods, modelling indicates that population level dietary adequacy can be achieved for eight of the 12 nutrients modelled. These choices include cows' milk, legumes, green leafy vegetables, sweet potatoes and fruits. Overall results suggest these high percentages of children at risk of inadequate nutrient intakes can be reduced through behaviour change interventions, although additional interventions may be required to ensure population‐level dietary adequacy for iron, thiamine and niacin.

## Key messages

- Dietary adequacy, for breastfed 12–23‐month‐old rural Eastern Ugandan children, is poor with over 45% of children at risk of inadequate intakes of nine micronutrients.
- Inadequate intakes of micronutrients likely contribute to growth faltering in this population where 21% of children are stunted but less than 4% are wasted.
- Dietary adequacy can be improved through careful selection of nutrient‐dense foods, including cows' milk, legumes, green leafy vegetables, sweet potatoes, and fruits.
- A food‐based approach alone, however, will not reduce the percentage of children at risk of inadequate intakes to low levels, for iron, thiamine and perhaps niacin, indicating alternative interventions are required.

## 1 INTRODUCTION

Children up to 2 years of age, are prone to under‐nutrition, because of their high energy and micronutrients requirements to support rapid rates of growth and development. The quality of complementary feeding (CF) diets, breastfeeding, infant care practices, food security, and health are key determinants of nutritional status at this age (Black et al., 2008; UNICEF, 2013). Optimal CF practices have been associated with a 5%–20% reduction in childhood stunting and up to 6% reduction in childhood deaths underscoring the importance of good CF practices in early life (Black et al., 2008). Nevertheless, young children, in low‐and‐middle‐income countries, are often fed complementary foods of low nutrient density that do not meet their nutrient requirements (PAHO, 2001; Shrimpton et al., 2001).

In Uganda, the 2016 Demographic and Health Survey (DHS) shows 53%, 29% and 4% of 6–59‐month‐old children are anaemic, stunted and wasted, respectively, and only 15% of 6–23‐month‐old children are fed a minimum acceptable diet (UBOS, 2018). Even though these rates of stunted growth are high, they have declined from 38% in 1994 to 29% in 2016, which has been attributed in part to national efforts promoting nutrition interventions in the first 1000 days of life (Buzigi, 2018). This annual rate of decline, however, is too slow to achieve the 2025 World Health Assembly Global Nutrition targets, for Uganda, to reduce stunting by 40% (Buzigi, 2018).

To improve the quality of CF diets, the World Health Organisation (WHO) promotes locally available and produced foods that are safe, acceptable, affordable and sustainable. Commercial products are discouraged, unless they fill critical nutrient gaps in local CF diets and complement rather than replace breast milk and nutrient‐dense locally produced foods (WHO, 2013). Among older preschool‐aged children, in Kampala, South‐west and North Uganda, inadequacy of iron, calcium, zinc, vitamins A and B12 are common (Harvey et al., 2010). Younger children are at even higher risk of inadequate micronutrient intakes than older children given their more rapid growth rate (Dewey & Brown, 2003), indicating an urgent need to improve the quality of CF diets.

Nevertheless, little is known about the nutrient adequacy of CF diets fed to young children, in Uganda, and the extent to which it could be improved through the selection of locally available foods. This study, therefore, aimed to estimate the percentage of breastfed 12–23‐month‐old rural Eastern Ugandan children at risk of inadequate nutrient intakes and to identify promising food choices for improving dietary adequacy.

## 2 METHODS

### 2.1 Study design and sampling

A cross‐sectional survey was conducted between January and February 2018 (dry season) in Bugiri and Kamuli Districts, Eastern Uganda. In this study, dietary, anthropometric and socioeconomic data were collected. Problem nutrients (i.e., where requirements will be difficult to meet given dietary practices) and food choices to improve dietary adequacy were identified, using linear programming analyses.

The sampling frame consisted of lists of all mothers with a child aged between 12 and 23 months who were living in 22 purposefully selected communities that were participating in the Sasakawa Global 2000 Uganda (SG2000 Uganda) country programme. This programme, in Uganda, focuses on transferring improved agricultural technologies to small holder families to enhance household incomes and food security. These lists were compiled by the SG2000 community‐based facilitators. Twelve mother–child pairs in each village were randomly selected to participate in the study. Substitutions were made when a pair did not meet the inclusion/exclusion criteria. Mother–child pairs were excluded if the child was less than 12 months or greater than 23 months of age, was not yet eating solid foods on a regular basis, was a multiple‐birth child, the mother was unable to communicate in Lusoga, Luganda or English, either the mother or child had a severe disability, the mother was not the biological mother of the child, the mother was a co‐wife with another mother selected to participate in the study or either the mother or child was not available for the duration of the study. For the current study, only the subsample of breastfed children were included in the analyses, to provide food‐based recommendations (FBRs) that align with the Ugandan government's policy on infant feeding (MOH, 2009).

Ethical approval was obtained from the Uganda National Council for Science and Technology, the London School of Hygiene & Tropical Medicine Observational Research Ethics Committee, and the University of Greenwich Faculty of Engineering and Science Ethics Committee. Written informed consent or thumb print was obtained from all mothers who participated in the study.

### 2.2 Data collection

Data on sociodemographic characteristics, housing conditions and water, sanitation and hygiene were collected one day before the day of dietary data collection via an interviewer‐administered questionnaire. Duplicate, serial (i.e., all maternal and child measurements were done once and then they were done a second time) anthropometric measurements of weight and height/length were taken of the mothers and children to the nearest 0.1 kg and 0.1 cm.

Food and beverage consumption of each child was estimated using a 1‐day weighed food record (WFR) where the enumerator weighed all foods and beverages consumed by the child using a dietary scale (±1 g; Salter Disc Electronic Digital Scale Model 1036). The enumerator also collected recipe data by weighing raw recipe ingredients and the final cooked food, and the cooking methods were recorded. If the child was left in the care of a person other than the mother, the enumerator remained with the mother; and on the child's return, she asked the secondary caregiver to recall any foods or drinks consumed by the child while in their care. The amounts of any foods/beverages consumed by the child before 07:00 or after 21:00 were also recalled and recorded. These recalled amounts were estimated by weighing equivalent amounts of playdough, dry rice, water, or the actual food (of a similar size to that consumed), depending on the food. These weights were converted to the grams of food consumed using conversion factors developed by Harvest Plus.

### 2.3 Data analysis

*Z*‐scores for each child's weight‐for‐length and length‐for‐age were calculated using the WHO 2006 Growth Standards (WHO, 2006). The body mass index of each mother was also calculated (weight/height^2^).

Dietary diversity scores were calculated based on the seven food groups previously recommended by the WHO (WHO, 2010). The minimum dietary diversity scores (MDDS) was defined as a diet that included foods from at least four of these seven food groups (WHO, 2010). This metric of dietary diversity was used instead of the more recent WHO definition based on eight food groups (WHO, 2021) to allow comparisons with the literature. The estimated percentage of children achieving a MDDS would not change if the more recent metric had been used because all children were breastfed.

To estimate each child's energy and nutrient intakes, a food composition table (FCT) was developed based on the Harvest Plus Ugandan FCT (Hotz et al., 2012), Optifood's internal FCT (Hotz et al., 2011) and the United States Department of Agriculture (USDA) National Nutrient Database for Standard Reference (Haytowitz et al., 2011). Nutrient contents for each cooked food ingredient, were adjusted for cooking losses, using the USDA retention factors, when only raw ingredient FCT were available (USDA, 2007). These data together with the food consumption data were used to estimate the daily intakes of energy and nutrients from complementary foods. Total daily intakes of energy and nutrients (i.e., from breast milk and diets) were estimated, assuming each child consumed 541 g/day of breast milk. This quantity was the median difference of estimated energy requirements and estimated energy intakes from complementary foods, assuming the energy content of breast milk was 66 kcal/100 g. Energy requirements were calculated using the FAO/WHO human energy requirements equation (FAO/WHO/UNU, 2004).

The percent of children at risk of inadequate nutrient intakes was estimated using the Intake Modelling Assessment and Planning Program software (IMAPP version 1; Iowa State University, 1995) after adjusting the intake distributions using IMAPP's internal variance ratios (NHANES 2003–2008) and its internal harmonised estimated average requirements. A sensitivity analyses was done to examine the effect of using the internal NHANES variance ratios by rerunning the analyses four times using one of the following variance ratios for each analysis: 0.2, 0.5, 0.65 or 0.8.

The nutrient densities of the CF diets (nutrient content per 100 kcal) were compared with desired levels (Dewey & Brown, 2003).

All statistical analyses were done using Stata IC version 16.1; and depending on the shape of the distributions, either means and standard deviations or median and quartiles were calculated.

### 2.4 Linear programming analyses

Linear programming was used to generate and test FBRs, identify problem nutrients and identify the best food sources of nutrients from locally consumed foods. Twelve nutrients (protein, iron, calcium, zinc, niacin, riboflavin, thiamine, folate, and vitamins A, B12, B6 and C) were modelled. All analyses were done in Modules II and III of the Optifood software, as described in detail elsewhere (Daelmans et al., 2013).

Foods modelled included breast milk, the foods consumed by ≥5% of breastfeeding children and less frequently consumed nutrient‐dense foods, including fruits (mangoes, oranges, papaya), vegetables (amaranth leaves, kale, and cabbage), eggs, yellow flesh sweet potatoes and sesame paste (Table S1).

The serving size modelled, for each food, was its WFR median serving size among consumers (g/meal) after adjusting, for consistency, the serving sizes modelled of similar foods (Table S1). For example, the serving size of brown sugar and white sugar were both modelled as 7 g/meal even though the observed median serving size of the less frequently consumed brown sugar (i.e., 13 times for brown sugar vs. 65 times for white sugar) was 11 g/meal. To increase the number of observations used to calculate the serving size modelled for each food, the WFR data collected from both breastfed and nonbreastfed children, in this survey, were used.

The model constraints, which ensured all Module II and III modelled diets were realistic, included constraints on the minimum/maximum number of servings per week from individual foods, food groups or food subgroups, as well an equality constraint on the diet's energy content (equal to the children's average energy requirements). The minimum and maximum constraint levels on the number of foods from different food groups and food subgroups were the 10th and 90th percentiles observed, for breastfed children, multiplied by seven to simulate a 7‐day diet (Table S2).

In Module II, two diets were modelled. The objective function, for both models, included nutrient goals which aimed to select a diet that came as close as possible to meeting the WHO/FAO recommended nutrient intakes (RNIs) for all nutrients (FAO/WHO Joint Report, 2004; WHO/FAO/UNU Consultation Report, 2007), except zinc, for which the International Zinc Nutrition Consultative Group RNI was used (Hotz & Brown, 2004). The assumed bioavailability for iron and zinc was low. In one of these two diets, the objective function included additional goals on food group patterns that aimed to select a diet that also came as close as possible to the observed median food group patterns of the breastfed children (Table S2). This diet represented the population's nutritionally best ‘average’ diet, whereas the other diet represented the nutritionally best diet for this population.

The food group patterns and main food sources of nutrients of the nutritionally best Module II diet (i.e., only nutrient goals) were examined to identify FBRs to test in Module III. These individual FBRs were expressed as the number of servings of foods from different food groups or food subgroups.

In Module III, 12 modelled diets were simulated that had the highest nutrient content (maximised), for each of 12 nutrients (‘best‐case scenario’ diets). These diets defined the problem nutrients (i.e., nutrients <100% RNI). Subsequently in Module III, for each individual FBR or set of FBRs tested, 12 model diets were simulated that had the lowest nutrient content (minimised) for each of 12 nutrients (‘worst‐case scenario’ diets). For these simulations, individual FBRs were initially screened and eight were selected to systematically combine and test. In this systematic analysis, all combinations of the individual FBRs (i.e., each pair, each combination of three FBRs, etc.) were analysed providing up to 247 different combinations. Results of these systematic analyses were then examined to identify sets of FBRs that would ensure at least 65% of the RNI for the highest number of nutrients.

One final series of analysis was run to determine whether doubling the quantity of one or two FBRs (doubling a food's serving size or the maximum constraint on the number of servings per week from a food group or food subgroup) would improve population‐level dietary adequacy. To identify these modifications, each food's nutrient content was expressed per average serving size, per maximum grams per week (average meal‐based serving size × maximum number of servings per week) and per 1000 kcal to identify, for nutrients that remained below 65% of their RNI in the Module III analyses, their best food sources. After modifying the model parameters, the Module III analyses were repeated.

Finally, to evaluate and compare the acceptability of selected individual and sets of individual FBRs, the percentage of children consuming at least one serving, for each individual FBR, were calculated.

## 3 RESULTS

**Figure 1:**
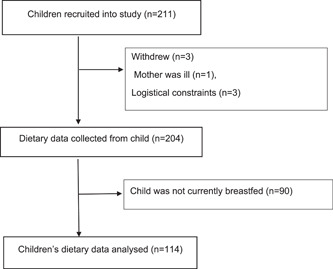
Selection of children, loss to follow‐up and exclusions

Of the 206 children who participated, 91 children were excluded from the current analyses because they had stopped breastfeeding, providing a final sample size of 114 breastfed children for the analyses (Figure 1). Their sociodemographic, anthropometric and dietary characteristics are presented in Table 1. Close to one quarter of these children were stunted but less than 4% were wasted; and just over one‐half of these children were consuming diets that met the MDDS of four food groups (Table 1).

**Table 1:** Sociodemographic and anthropometric characteristics of the participants

	*n* a	Mean (SD)	*n* (%)
Child's age (months)	113	16.0 (2.7)	
Child's sex, male	114		60 (52.6)
Child's length‐for‐age *Z*‐score	113	−1.19 (1.23)	
% stunted^b^			24 (21.2)
Child's weight‐for‐length *Z*‐score	106	−0.29 (1.03)	
% wasted^c^			4 (3.8)
Child's dietary diversity score	113	3.58 (0.9)	
% ≥4 food groups			61 (54.0)
Maternal age (years)	113	26.8 (6.2)	
Relationship status	112		
Monogamous relationship			68 (60.7)
Polygamous relationship			32 (28.6)
Single, widowed, not in relationship			12 (10.7)
Maternal education	113		
None			6 (5.3)
Primary, uncompleted			62 (54.9)
Primary, completed			15 (13.3)
Secondary, uncompleted			25 (22.1)
Secondary completed/tertiary			5 (4.4)
Maternal body mass index (kg/m^2^)	100	22.0 (2.8)	
Body mass index < 18.5 kg/m^2^			7 (7.0)
Body mass index > 25 kg/m^2^			13 (13.0)
Number of household members	113	6.5 (2.6)	
House roofing materials	113		
Grass thatch			35 (31.0)
Iron sheets			78 (69.0)
House wall materials	113		
Mud bricks			58 (51.3)
Cement bricks			55 (48.7)
Toilet facilities	112		
No toilet			1 (0.9)
Uncovered pit latrine			82 (73.2)
Covered pit latrine			26 (23.2)
Ventilated improved pit latrine/Flush			3 (2.7)

^a^ n, number of participants.

^b^ Stunted = length‐for‐age *Z*‐score <‐2SD.

^c^ Wasted = weight‐for‐length *Z*‐score <‐2SD.

The median nutrient densities of the CF diets were below desired levels for all nutrients except protein, and vitamins A, C and B6 (Table 2). Similarly, over 45% of children were at risk of inadequate intakes of all nutrients, except protein, vitamin A and vitamin C; with >90% at risk of inadequate intakes of iron, calcium and niacin (Table 2).

**Table 2:** The median (quartiles) daily intakes of energy and nutrients from complementary foods alone and from both complementary foods and breast milk,^a^ the median (quartiles) nutrient density of complementary foods compared with desired densities, and the percentage of children at risk of inadequate intakes of nutrients (*n* = 114)

	Daily intakes from complementary foods alone	Nutrient density of CF (per 100 kcal)	Desired nutrient densities^b^	Total daily intakes	% At risk of inadequate intakes^c^
Energy (kcal)	385 (273, 574)	NA	NA	742 (629, 935)	NA
Protein (g)	8.3 (5.8, 13.8)	2.2	0.9	13.8 (11.3, 19.4)	16.8
Iron (mg)	2.0 (1.3, 2.9)	0.5	1.2	2.1 (1.4, 3.0)	100.0
Zinc (mg)	1.3 (0.9, 2.0)	0.3	0.4	1.9 (1.4, 2.6)	54.7
Calcium (mg)	65 (34, 127)	17	63	211 (179, 274)	97.0
Thiamine (mg)	0.2 (0.1, 0,3)	0.05	0.07	0.3 (0.2, 0.4)	79.9
Riboflavin (mg)	0.2 (0.1, 0.3)	0.04	0.06	0.3 (0.3, 0.5)	57.2
Niacin (mg)	1.9 (1.2, 3.2)	0.5	0.9	2.7 (2.0, 4.0)	91.5
Folate (µg DFE)	42 (27, 64)	11.4	19	86 (70, 108)	81.7
Vit B12 (µg)	0.3 (0.0, 0.8)	0.07	NA	0.79 (0.49, 1.26)	46.1
Vit B6 (mg)	0.3 (0.2, 0.5)	0.08	0.08	0.4 (0.2, 0.6)	51.3
Vitamin A (RAE)	37 (18, 81)	9.4	5	302 (283, 346)	0.0
Vitamin C (mg	11.7 (5.3, 27.3)	3.4	0	32.8 (26.4, 48.9)	0.0

Abbreviation: CF, complementary food; DFE, mcg of dietary folate equivalents; RAE, mcg of retinol activity equivalents.

^a^ Assumed each child consumed 541 g/day of breast milk. The energy and nutrient content per 100 g of breast milk was: energy = 66  kcal, protein = 1.02 g, Fe = 0.03 mg, Zn = 0.1 mg, Ca  = 27 mg, thiamine = 0.02 mg, riboflavin = 0.034 mg, niacin = 0.146 mg, folate = 8 µg DFE, vitamin B12 = 0.09 µg, vitamin B6 = 0.011 mg, vitamin A = 49 µg, RAE and vitamin C = 3.9 mg.

^b^ New DRI average desired density, for iron and zinc, assumes moderate bioavailability (Dewey & Brown, 2003).

^c^ Estimated using IMAPP software, assuming low bioavailability for iron and zinc and IMAPP's internal variance ratios. The harmonised estimated average requirements (EAR) used in IMAPP are: protein = 11 g, Zn = 2.0 mg, Ca = 400 mg, thiamine = 0.4 mg, riboflavin = 0.4 mg, niacin = 5.0 mg, folate = 120 DFE, vitamin B12 = 0.7 µg, vitamin B6 = 0.4 mg, vitamin A = 201 RAE and vitamin C = 13 mg. The percentage at risk of inadequate iron intakes was estimated using the full probability approach.

In total, 121 foods were consumed, however, only 38 of these foods were consumed by ≥5% of the children. Most children consumed grain and grain products and starchy roots and tubers. About a third of the children consumed fresh cow's milk and 45% consumed small fish with bones (mukene; silver cyprinid or Lake Victoria sardine, Rastrineobola argentea). Consumption of other animal source foods was low. Similarly, consumption of fruits and vegetables was low with only jackfruit, tomatoes and onions being consumed by more than 15% of the children (Table S1).

In Optifood, 49 foods were modelled, including 38 commonly consumed foods and 11 rarely consumed nutrient‐dense foods, including five vegetables, two fruits, eggs, sesame paste and yellow‐flesh sweet potatoes. Only 16 of these 49 foods had a median serving size above 20 g/meal (Table S1).

The Module II nutritionally best diet did not achieve RNIs for six nutrients (Table 3). Of these nutrients, only calcium, thiamine, niacin and iron remained ‘problem nutrients’, because 100% of the zinc and folate RNI were achieved, when their nutrient contents were maximised in Module III; indicating their RNI's could be met using locally available foods but to the detriment of achieving the RNIs of other nutrients (Table 3).

**Table 3:** The nutrient content of the Module II optimised diets and the Module III maximised (without recommendations) and minimised diets tested without food‐based recommendations and with selected food‐based recommendations expressed as a percentage of their recommended nutrient intakes^a^

	Ca%	Vit C%	Vit B1%	Vit B2%	Niacin%	Vit B6%	Folate%	Vit B12%	Vit A%	Fe%	Zn%	≥65%^b^
Optimised diet_average^c^	49	174	66	82	56	81	78	216	100	30	79	NA
Optimised diet_best^d^	76	192	77	121	64	100	96	314	100	30	88	NA
Maximised^e^	83	231	84	129	72	128	100	317	123	37	101	NA
Minimised^e^	30	70	42	45	28	31	37	55	66	10	43	2
Frt14_ Leg14_ Milk14_SPot7	65	91	50	92	34	58	66	97	98	18	65	7
Frt7_Leg14_ MFE7_Spot7_WGrt14	43	109	61	65	46	68	67	160	93	27	84	7
Frt14_Milk7_ SPot7_WGrt14	51	90	56	78	38	66	48	76	95	22	77	6
Frt14_ Leg14_ Milk7_ WGrt14	48	86	55	71	37	57	66	76	71	25	82	6
Frt14_MFE7_SPot7_WGrt14	40	90	54	66	41	68	47	160	93	23	73	6
Milk7_SPot7_WGrt14	48	76	52	72	34	56	42	76	93	20	74	5
Frt7_Leg7_MFE7_Veg21_SPot7_WGrt14	39	83	54	63	41	65	50	160	92	23	76	5
Leg14_MFE7_Veg21_SPot7_WGrt14	40	78	57	63	42	65	61	160	91	27	83	5
Double quantity of milk and green leafy vegetables												
Frt14_GLV4_ Leg14_ Milk14_SPot7	95	121	57	130	36	67	73	137	120	20	79	8
GLV4_Leg14_MFE7_ Spot7_WGrt14	47	106	59	68	44	72	66	160	108	30	87	7

Abbreviations: Frt7, 7 servings of fruits per week; Frt14, 14 servings of fruits per week; GLV4, 4 servings of green leafy vegetables per week; Leg7, 7 servings of legumes per week; Leg14, 14 servings of legumes per week; MFE7, 7 servings of meat, fish or eggs per week; Milk7, 7 servings of milk per week; Milk14, 14 servings of milk per week; Spot7, 7 servings of sweet potatoes per week; Veg21, 21 servings of vegetables per week; Vit, vitamin; Wgrt14, 14 servings of whole‐grain cereals per week.

^a^ WHO recommended nutrient intakes for all nutrient except zinc (FAO/WHO/UNU, 2004; WHO/FAO/UNU Consultation Report, 2007). For zinc it was the iZinCg recommended nutrient intakes (Hotz & Brown, 2004). The RNIs used were: Ca = 400 mg/day; vitamin C = 30 mg/day; thiamin = 0.3 mg/day; riboflavin = 0.4 mg/day; niacin = 4 mg/day; vitamin B6 = 0.3 mg/day; folate = 80 µg DFE; vitamin B12 = 0.7 µg/day; vitamin A = 400 µg RE/day; Fe = 9.3 mg/day; Zn = 3 mg/day.

^b^ The number of nutrients that were ≥65% of their RNI in their Module III minimised diet.

^c^ Module II optimised diet with both nutrient and food pattern goals.

^d^ Module II optimised diet with only nutrient goals.

^e^ Maximised—the 11 Module III diets in which the objective function maximised the content of each nutrient; when a food‐based recommendation was not tested; minimised—the 11 Module III diets in which the objective function minimised the content of each nutrient, when a food‐based recommendation was not tested.

The food patterns of the two Module II diets show that dietary adequacy can be improved by increasing the number of servings per week of fruits, vegetables, dairy products, roots–tubers, meat, fish and eggs (MFE) and legumes, while decreasing the number of servings per week from added sugar, fat and cereal‐based staples compared with the median number of servings consumed (Table S2). The best food sources of nutrients in these Module II diets were breast milk, sweet potatoes, millet flour, cows' milk, mukene, kidney beans, kale and papaya, (Table S3). Based on these results, the individual FBRs initially screened in Module III included alternative numbers of servings per week from these six food groups, as well as from eight food subgroups (green leafy vegetables, beans/lentils, nuts/seeds, small fish with bones, eggs, unrefined grains, vitamin‐C‐rich fruits and vitamin‐A‐rich roots/tubers) and millet flour (Table S4).

Based on these results, 11 individual FBR were selected to systematically combine and test, in three series of analyses of eight FBRs per systematic analyses. These FBRs were 7 or 14 servings per week of dairy products, legumes, or fruit, 7 servings per week of MFE or vitamin A‐rich roots/tubers, 21 servings per week of vegetables, 14 servings per week of whole‐grain cereals and 2 servings per week of green leafy vegetables. Overall, only 383 unique sets of FBRs were evaluated, because some sets were unfeasible, that is, they exceeded the energy constraint (Table S5). From these results, eight sets of FBRs were selected as the most promising for improving dietary adequacy (Table 3). However, their adoption would only ensure population‐level dietary adequacy for between five to seven micronutrients, depending on the set of FBRs. In all sets, the ‘worst‐case scenario’ analyses for calcium, thiamine, niacin, and iron remained below 65% of the RNI (Tables 3 and S5).

The best food sources of these four nutrients, whether expressed per 100 kcal, per serving size or per maximum grams per week were whole grain cereals, legumes, vegetables, roots and tubers, cows' milk, mukene and jackfruit (Table S6). When expressed per 100 kcal, cows' milk and green leafy vegetables were good food sources for the highest number of nutrients and were thus chosen as the food sources to model at double the observed median quantities consumed in the repeat series of analyses. In these analyses, 317 sets of FBRs were assessed, and the number of nutrients meeting 65% of their RNIs, in the worst‐case scenario analyses, increased from 7 to 8 of 11 micronutrients modelled. Thiamine, niacin and iron remained below 65% of their RNIs, indicating alternative interventions are needed to ensure their dietary adequacy at the population level, especially for iron (Table 3).

Of the individual FBRs selected for systematic testing, fruits, vegetables and unrefined cereals were consumed by over 75% of children, and sweet potatoes, legumes and MFE were consumed by over 50% of children on the WFR day. Less than one‐quarter of children consumed cows' milk or green leafy vegetables (Table 4). When individual FBRs were combined into sets of four or more FBRs, approximately one‐fifth of children consumed fruit, MFE, sweet potatoes and whole‐grain cereals on the WFR day, however, this set had low ‘worst‐case‐scenario’ calcium and folate levels compared with alternative sets (Table 3). Only those sets including cows' milk and legumes had ‘worst‐case scenario’ values, for calcium and folate, ≥65% of their RNIs (Tables 3 and 4). Of these sets, the set with recommendations for consumption of fruits, legumes, milk and sweet potatoes was the most promising, as 7.4% of children were consuming foods from each of these food groups/subgroups and the worst‐case scenarios for seven micronutrients were ≥65% of their RNIs when the milk serving size was the median serving size observed. Less than 1% of children consumed foods from the set of FBRs, including both green leafy vegetables and cows' milk, which ensured population‐level dietary adequacy for 8 of the 11 micronutrients modelled.

**Table 4:** Number and percentage of children consuming^a^ at least one serving of individual food‐based recommendations and of each set of food‐based recommendations selected

Individual FBRs	*n*	% Consumers	Sets of FBRs selected	*n*	% Consumers
Cereals, unrefined	180	88.2	Frt_GLV_ Leg_Milk_SPot	1	0.5
Fruits	158	77.5	Frt_Leg_MFE_SPot_Veg_WGrt	19	9.3
Green leafy vegetables	11	5.4	Frt_Leg_MFE_Spot_WGrt	20	9.8
Legumes	118	57.8	Frt_Leg_Milk_SPot	15	7.4
Meat, fish or eggs	115	56.4	Frt_Leg_Milk_WGrt	0	0
Milk	49	24.0	Frt_MFE_SPot_WGrt	42	20.6
Sweet potatoes	106	52.0	Frt_Milk_SPot_WGrt	0	0
Vegetables	182	89.2	GLV_Leg_MFE_Spot_WGrt	1	0.5
			Leg_MFE_SPot_Veg_WGrt	22	10.8

Abbreviations: FBR, food‐based recommendation; Frt, fruits; GLV, green leafy vegetables; Leg, legumes; MFE, meat, fish or eggs; Spot, sweet potatoes; Veg, vegetables; Wgrt, whole grain cereals, unrefined.

^a^ In the 1‐day weighed food record.

## 4 DISCUSSION

This study shows poor nutritional adequacy of CF diets consumed by children 12–23‐months in rural Eastern Uganda. The nutrient densities of CF diets were below desired levels for 7 of the 12 nutrients examined. Over 45% percentage of children were at risk of inadequate intakes for 8 of the 12 nutrients examined with inadequate intakes of calcium, iron and niacin being over 90%. In contrast, energy intakes of these children appear adequate, given the low percentage of children who were wasted. Together these results suggest that poor quality CF diets are common in the region, indicating a need to improve the quality of CF diets given the negative functional consequences of micronutrient under‐nutrition (Best et al., 2011; Black et al., 2008; Dewey, 2013; Geng et al., 2018; Zhao et al., 2017).

Both low dietary diversity and small serving sizes of nutritious foods contribute to poor quality CF diets. Approximately half of the children consumed less than four nutritious food groups per day; and median serving sizes of nutrient‐dense animal source foods, such as small dry fish, eggs, and milk, were only 2, 4, and 52 g per serving, respectively. The servings sizes of small dry fish and eggs were small because they are usually consumed as an ingredient in a sauce. Eggs were also rarely consumed (only 4% of children). Similarly, nutrient‐dense vegetables, such as carrots, pumpkins or green leafy vegetables were rarely consumed. Thus, to improve the quality of CF diets, both an increase in food diversity and their serving sizes will be required.

Diet modelling confirmed the above findings. The Module II nutritionally best diets show that, to improve the nutrient content of these CF diets, both a reduction in the number of servings of staple foods, and a twofold or more increase from the average amounts of milk, legumes, and vegetables consumed, is required. The Module III analyses, in which 383 alternative sets of food‐based choices were evaluated, indicate that even though feeding the recommended minimum number of four nutritious food groups will improve dietary adequacy, the right combinations of these four food groups are essential and the food serving sizes need to be large. Regarding quantities, the number of servings of fruit, milk and legumes selected were at the observed 90th percentiles of intakes, and doubling the average serving sizes of milk and green leafy vegetables further improved the modelled population‐level dietary adequacy. Only iron, thiamine and niacin remained below desired levels in all sets of food‐based choices evaluated.

Among the best sets of four food group combinations identified, feeding the children 2 servings per day of milk, legumes and fruit and one serving per day of sweet potatoes would reduce the high percentage (46%–97%) of children at risk of inadequate intakes of vitamins B6 and B12, zinc, calcium, riboflavin and folate. Comparatively, this set of food‐based recommendations was also realistic. On the WFR day, milk, legumes, fruit and sweet potatoes were consumed by 24%, 58%, 78% and 52% of children, respectively; and 7.4% of children consumed foods from all four of these groups. Only one set among those selected aligned more closely with the observed food consumption patterns (21% of children consumed fruit, MFE, sweet potatoes and whole‐grain cereals on the WFR day). However, this set did not achieve a worst‐case scenario dietary calcium content indicative of population‐level adequacy (worst‐case scenario was only 40% of the calcium RNI). The other two sets of four food groups selected did not align closely with the children's observed food consumption patterns.

These modelling results provide insights into why a high percentage of children are at risk of inadequate intakes of multiple nutrients, even though over half of the children were consuming diets that met the recommended MDDS of four or more food groups (WHO, 2010). In this region, even though consuming foods from four nutritious food groups is adequate to improve dietary adequacy, for most nutrients, it is insufficient. The results indicate that both the selection of specific combinations of the four food groups and the quantity of nutritious foods consumed are important.

These results are not unique to the Eastern region of Uganda. A dietary survey among slightly older children (24–59 months), in Kampala, North and South‐West Uganda, found that over 30% of children were at risk of inadequate intakes of calcium, iron, zinc, vitamin B12 and vitamin A; and depending on the region, over 15% of children were at risk of inadequate intakes of thiamine, riboflavin and folate (Harvey et al., 2010). Another dietary survey in Western Uganda found a lower percentage of 1‐year old children were at risk of inadequate intakes of iron (14% vs. 100%) and zinc (7% vs. 55%), and a higher percentage of these children were at risk of inadequate intakes of protein (43% vs. 17%) and vitamin A (41% vs. 0%) than in our study (Isingoma et al., 2017). Regional differences and differences in dietary assessment methodology, methods of analyses and the criteria used to define adequate nutrient intakes might account for these differences between the current and earlier Western Ugandan study. In contrast, the percentage of children consuming diets that met the recommended MDDS of four food groups was similar for the two studies (48% vs. 54%), although both estimates were higher than reported, for 12–23‐month‐old children, in the 2016 Ugandan DHS (i.e., 32%) (Isingoma et al., 2017; UBOS, 2018).

Our findings are also consistent with modelling results from similar studies conducted in rural Kenya and Ethiopia which suggest that modifying food choices within the limits of local CF practices will not achieve population‐level dietary adequacy for calcium, riboflavin, folate, iron, zinc, and/or niacin, depending on the setting (Ferguson et al., 2015; Samuel et al., 2019; Talsma et al., 2017; Vossenaar et al., 2016). They also align with CF recipes developed, for 12–23 m old children in the Eastern Region of Uganda, which included two or more of the following ingredients: millet, sweet potato, beans, groundnuts, small fish, green leafy vegetables and milk (Bekele & Turyashemererwa, 2019). In this previous study, Bekele and Turyashemererwa (2019) found the recipes were feasible and acceptable, if ingredients were available, because they were familiar to caregivers.

Our study, however, indicates that to ensure population‐level dietary adequacy, multiple intervention strategies are needed. Behaviour change communications strategies will improve dietary adequacy, for most nutrients, although encouraging an increase in the quantity of cows' milk and legumes fed to young children may come at a cost that some households cannot afford. Its success will also depend on the development of effective promotion materials and processes/structures of delivery. Nevertheless, even with an effective behaviour change strategy, the modelling results indicate a relatively high percentage of children would remain at risk of inadequate intakes of iron, thiamine, and niacin. Of these nutrients, iron is of highest concern, given the high prevalence of anaemia among young children in Uganda (UBOS, 2018), and our results showing the maximum iron content achievable in any modelled diet was only 37% of its RNI. In contrast, niacin is not a concern, because contributions from tryptophan were not modelled; and pellagra is not documented in Eastern Uganda. Similarly, there is no evidence of thiamine deficiency disorders among young children in Eastern Uganda; although the high percentage of children at risk of inadequate intakes of thiamine suggest a biomarker study is warranted (Whitfield et al., 2018).

Other intervention strategies, to improve dietary iron and thiamine adequacy, include introducing and promoting the consumption of locally produced lipid‐based nutrient supplementation (LNS), commercially fortified toddler foods or point‐of‐use multiple micronutrient powders. In Western Uganda, LNS improved dietary adequacy among 6–59‐month‐old children enrolled in a 10‐week community‐based programme; although low levels of consumption were observed in both this trial and others conducted elsewhere (Ickes et al., 2015). Similarly, children in the current study were consuming a limited number of commercial food products, which might limit the success of a food fortification programme.

Alternatively, fortification through point‐of‐use multiple micronutrient powders has been associated with an increase in height, haemoglobin concentration, plasma/serum concentrations of ferritin, retinol, zinc, and a decrease in soluble transferrin receptor concentration, although except for anaemia, the results have been mixed (Tam et al., 2020). This strategy could also have unintended negative effects via an increase in iron‐induced morbidity (Brittenham, 2012; Zimmermann et al., 2010). In the long term, agriculture interventions that increase access to micronutrient‐rich foods combined with effective behaviour change communication should be considered, including through value addition, biofortification, small animal production or kitchen/community gardening.

One of the strengths of this study was the use of WFRs to estimate intakes of energy and nutrients. It is the gold standard method of dietary assessment because data are collected prospectively, and the amounts consumed are weighed and recorded at the time of consumption (Gibson, 2005). A second strength was the use of mathematical optimisation to identify the nutritionally best diets and objectively evaluate over 300 alternative sets of food‐based combinations. These robust analyses simultaneously consider multiple nutrients and local food consumption patterns.

Study weaknesses include the collection of dietary data on only 1 day and in one season, which limits the accuracy of weekly food pattern model constraints and the generalisability of results to other seasons. It also meant, when estimating the percentage of children at risk of inadequate intakes in IMAPP, American variance ratios were used to adjust the intake distributions for intrasubject variability. A sensitivity analyses, however, showed that except for protein and riboflavin, the influence on prevalence estimates of inadequate intakes is minor (Table S7). Other limitations, as in all dietary surveys, is the robustness of conclusions depends on the accuracy of the food composition data base. Further, some children consumed foods before the enumerator arrived at in the morning (07:00) or after they left in the evening (21:00). These foods were recalled, which might introduce a minor recall bias. The intakes of breast milk were estimated, and the same quantity of breast milk was incorporated into the diets of all children. This assumption that individual level errors of over‐ and under‐estimation of breast milk intakes would balance at the population level might be incorrect.

In conclusion, 1‐year old breastfed children from rural Eastern Uganda are at high risk of inadequate intakes of multiple micronutrients. Both low dietary diversity and small serving sizes of nutritious foods contribute to these inadequate dietary intakes of nutrients. Successful behaviour change communication strategies encouraging caregivers to feed young children servings of milk, legumes, green leafy vegetables, fruit, sweet potatoes, and whole grains will improve dietary adequacy. However, alternative intervention strategies are needed to achieve it for all nutrients, especially iron. These results also point to a need for a micronutrient biomarker survey and research on how to increase the availability and accessibility of nutrient‐dense foods and how to effectively promote realistic FBRs through behaviour change communication strategies.

## CONFLICT OF INTERESTS

The authors declare that there are no conflict of interests.

## AUTHOR CONTRIBUTIONS

KW, ELF and JN designed the research study. ALSB and JN were involved in data collection and study management. NCK, HL and ELF analysed the data. NCK and ELF wrote the manuscript. All authors read and approved the final manuscript.

## Supporting information

Supplementary Information

## Data Availability

The data that support the findings of this study are openly available in Harvard Dataverse https://doi.org/10.7910/DVN/J45QQP.
