# Supplementary material for: A food‐based approach could improve dietary adequacy for 12–23‐month‐old Eastern Ugandan children
Source: Matern Child Nutr. 2022 Jan 3;18(2):e13311. doi: 10.1111/mcn.13311 (PMC8932727; doi:10.1111/mcn.13311)
Supplement: Supplementary file 1 — Supplementary Information [file MCN-18-e13311-s001.docx]

**Online Supplementary Materials**

**Supplementary Table 1:** The median portion size**^+^** and the number and percentage of 12-23-month-old Eastern Ugandan children who had consumed each food that were modelled in Optifood

| **Food** | **Serving_O^+^ (g/meal)** | **Serving_M**‡  **(g/meal)** | **n** | **%** |
| --- | --- | --- | --- | --- |
| Breastmilk, human, mature (developing countries) § | N/A | 541 | 114 | 100.0 |
| **Added fats** |  |  | 84 | 73.7 |
| Vegetable Oil, Mukwano, fortified, raw (cooked) | 1 | 1 | 18 | 15.8 |
| Vegetable Fat, unspecified brand, raw (cooked) | 1 | 1 | 66 | 57.9 |
| **Added sugars** |  |  | 79 | 69.3 |
| Sugarcane, raw | 4 | 5 | 4 | 6.1 |
| Sugars, brown | 11 | 7 | 13 | 11.4 |
| Sugars, granulated | 7 | 7 | 65 | 56.5 |
| **Bakery & breakfast cereals** |  |  | 51 | 44.7 |
| Pancake, banana/cassava flour, fried | 15 | 15 | 8 | 7.0 |
| Bread, pita, white, fresh | 21 | 21 | 18 | 15.8 |
| Biscuit, sweet, plain, unfortified | 8 | 8 | 11 | 9.7 |
| Doughnut, basic recipe, fried | 15 | 16 | 19 | 16.7 |
| **Dairy products** |  |  | 30 | 26.3 |
| Milk, cow, whole, fresh, raw (boiled) | 52 | 52 | 35 | 30.7 |
| **Fruits** |  |  | 84 | 73.7 |
| Banana, ndiizi, ripe, fresh, raw | 24 | 29 | 7 | 6.1 |
| Jackfruit, ripe, fresh, raw | 29 | 29 | 61 | 53.5 |
| Plantain, unripe, pale flesh, boiled (without salt), drained | 30 | 29 | 6 | 5.3 |
| Mango, orange flesh, ripe, raw | 14 | 29 | 5 | 4.4 |
| Tamarind, dried, raw | 5 | 5 | 6 | 5.3 |
| Orange, fresh, raw | 15 | 29 | 6 | 5.5 |
| Papaya, fruit, ripe, raw | 58 | 58 | 3 | 2.6 |
| **Grains & grain products** |  |  | 98 | 86.0 |
| Maize flour, white variety, refined, raw (boiled) | 17 | 35 | 11 | 9.7 |
| Rice, white, polished, raw (boiled) | 19 | 25 | 9 | 7.7 |
| Rice, white (polished), fried in oil | 36 | 44 | 15 | 13.2 |
| Maize flour, white variety, unrefined, raw (boiled) | 35 | 35 | 75 | 65.8 |
| Maize flour, yellow variety, unrefined, raw (boiled) | 21 | 35 | 9 | 7.9 |
| Millet flour, raw (boiled) | 8 | 35 | 8 | 7.0 |
| **Legumes, nuts & seeds** |  |  | 67 | 58.8 |
| Beans, kidney(other), dried, raw (boiled) | 10 | 10 | 16 | 13.9 |
| Groundnuts, powder, raw (boiled) | 4 | 4 | 15 | 13.2 |
| Kulakula nut, powder, dried, raw (boiled) | 4 | 4 | 7 | 6.1 |
| Sesame seed, paste, raw (boiled) | 9 | 4 | 3 | 2.6 |
| Peanuts, all types, dry-roasted | 6 | 6 | 10 | 8.8 |
| Soybean, dried, roasted | 4 | 6 | 13 | 11.4 |
| **Meat, Fish & Eggs (MFE)** |  |  | 61 | 53.5 |
| Egg, chicken, whole, fresh, fried (boiled) | 4 | 10 | 4 | 3.5 |
| Fish, silver fish (mukene), dried, raw (boiled) | 2 | 5 | 30 | 26.3 |
| Fish, silver fish (mukene), dried, roasted (boiled) | 2 | 5 | 21 | 18.4 |
| **Starchy roots & other starchy plant foods** |  |  | 75 | 65.8 |
| Cassava flour, raw (boiled) | 26 | 35 | 10 | 8.8 |
| Cassava, fresh, raw (boiled) | 33 | 38 | 11 | 9.7 |
| Sweet potato, white, wo/skin, fresh, (boiled) | 69 | 69 | 47 | 41.2 |
| Yam, (ndaggu, baluggu, others), fresh, raw (boiled) | 38 | 38 | 7 | 6.1 |
| Cassava, fresh, fried | 48 | 48 | 5 | 4.4 |
| Sweet potato, yellow, wo/skin, fresh, steamed (boiled) | 12 | 69 | 3 | 2.6 |
| **Sweetened snacks & desserts** |  |  | 11 | 9.6 |
| Candies, hard | 5 | 5 | 9 | 7.7 |
| **Vegetables** |  |  | 101 | 88.6 |
| Onion, large bulb, fresh, raw (boiled) | 0.22 | 0.22 | 77 | 67.5 |
| Eggplant, thick & short (type3),w/skin, fresh, raw (boiled) | 10 | 10 | 8 | 7.0 |
| Tomato, fresh, semi-ripe, raw (boiled) | 5 | 5 | 65 | 56.5 |
| Tomato, ripe, fresh, raw (boiled) | 5 | 5 | 24 | 21.1 |
| Avocados, fresh, raw | 12 | 12 | 12 | 10.5 |
| Amaranth leaves, fresh, fried (boiled) | 3 | 25 | 1 | 0.9 |
| Kale (sukumawiki) leaves, fresh, raw (boiled) | 48 | 25 | 2 | 1.8 |
| Carrots, fresh, raw (boiled) | 2 | 5 | 1 | 0.9 |
| Pumpkin, mature, wo/skin, fresh, (boiled) | 13 | 13 | 1 | 0.9 |
| Cabbage, green, fresh, fried (boiled) | 13 | 25 | 5 | 0.9 |

**^+^** the median amount consumed per meal for children who consumed the food. The weights are raw weights of an ingredient, for example 2 g for “Fish, silver fish (mukene), dried, raw (boiled)” is 2 grams of dry fish.

‡ the amount per serving per meal modelled in Optifood

§ the daily quantity of breastmilk consumed was estimated by subtracting the mean energy from complementary foods from the estimated mean energy requirement of the children, assuming breastmilk’s energy content was 66 kcal/100g.

**Online Supplementary Table 2:** The Module II food pattern constraints and goals, and the number of servings from each food group selected in the two Module II diets**^+^**

|  | **Model constraints and goals** | | | **Module II Results^¥^** | |
| --- | --- | --- | --- | --- | --- |
| **Food Groups** | **Observed 10th percentile**‡ | **Observed 50th percentile**§ | **Observed 90th percentile**‡ | **Module II nutritionally best average diet** | **Module II nutritionally best diet** |
|  | Number of servings per week | | | | |
| Added fats | 0 | 7 | 14 | 7 | 0 |
| Added sugars | 0 | 7 | 14 | 7 | 0 |
| Bakery & breakfast cereals | 0 | 0.1 | 14 | 0.1 | 1.8 |
| Dairy products | 0 | 0.1 | 14 | 0.1 | 14 |
| Fruits | 0 | 7 | 21 | 7 | 12 |
| Grains & grain products | 0 | 14 | 28 | 14 | 5 |
| Human milk | 6.999 | 7 | 7.001 | 7 | 7 |
| Legumes, nuts & seeds | 0 | 7 | 14 | 7 | 14 |
| Meat, fish & eggs | 0 | 7 | 12 | 7 | 12 |
| Starchy roots & other starchy plant foods | 0 | 7 | 21 | 7 | 10 |
| Sweetened snacks & desserts | 0 | 0.1 | 2 | 0.1 | 0 |
| Vegetables | 0 | 14 | 42 | 14 | 42 |

**^+^**one day weighed food record data was multiplied by 7 days to generate these data

‡the 10th and 90th percentiles were the upper and lower constraints used for food groups in all modelled diets

^§^the 50^th^ percentile was the food pattern goals used in the Module II model; the value 0.1 represents an observed food pattern value of 0 serves per week because mathematically a 0 cannot be used in the models

¥The number of servings of foods from different food groups in the Module II nutritionally best average diet (objective function minimized deviations under the RNIs and both under and over the observed median food group pattern) and the nutritionally best diet (objective function minimized deviations under the RNIs )

**Online Supplementary Table 3:** The number of nutrients for which individual foods and food sub-groups contribute at least 5% of the nutrients in the Module II diets that aim to achieve both nutrient and food pattern goals or only to achieve food pattern goals

| **Module II diet aiming to achieve only nutrient goals** | | | | **Module II diet aiming to achieve both food pattern & nutrient goals** | | | |
| --- | --- | --- | --- | --- | --- | --- | --- |
| **Food subgroups** | | **Individual foods** | | **Food subgroups** | | **Individual foods** | |
| **Sub-groups** | **# Nut**^+^ | **Foods** | **# Nut** | **Sub-groups** | **# Nut** | **Foods** | **# Nut** |
| Breastmilk | 10 | Breastmilk | 10 | Breastmilk | 10 | Breastmilk | 10 |
| Other roots & tubers | 9 | Millet flour | 7 | Vit A source roots/tubers | 8 | Yellow sweet potato | 8 |
| Whole & unrefined grains | 7 | White sweet potato | 7 | Whole & unrefined grains | 7 | Millet flour | 7 |
| Fluid or powdered milk | 6 | Cows’ milk | 6 | Dark green leafy vegetables | 4 | Maize flour unrefined, white | 5 |
| Small whole fish with bones | 5 | Kidney beans, dry | 4 | Small whole fish with bones | 4 | Silver fish (Mukene) | 4 |
| Nuts & seeds | 5 | Yellow sweet potato | 3 | Other roots & tubers | 3 | Maize flour unrefined, y | 4 |
| Beans & lentils | 4 | Silver fish (Mukene) | 3 | Beans & lentils | 3 | White sweet potato | 3 |
| Dark green leafy vegetables | 3 | Kale | 2 | Vitamin C rich fruit | 2 | Kidney beans, dry | 3 |
| Vitamin A rich roots & tubers | 3 |  |  |  |  | Kale | 2 |
| Vitamin C rich fruit | 2 |  |  |  |  | Papaya | 2 |

^+^ number of nutrients for which this food or food sub-group provided ≥5% of the total content of that nutrient in the Module II modelled diets

**Online Supplementary Table 4:** The individual food-based recommendations initially tested in Module III compared to the Module II optimised diets and minimised diets without a food-based recommendation expressed as a percentage of recommended nutrient intakes^+^

|  | **Ca**  **%** | **Vit**^‡^ **C**  **%** | **Vit B1**  **%** | **Vit B2**  **%** | **Niacin**  **%** | **Vit B6**  **%** | **Folate**  **%** | **Vit B12**  **%** | **Vit A**  **%** | **Fe**  **%** | **Zn**  **%** | **≥65%**^§^ |
| --- | --- | --- | --- | --- | --- | --- | --- | --- | --- | --- | --- | --- |
| Optimised diet_average^¥^ | 49 | 174 | 66 | 82 | 56 | 81 | 78 | 216 | 100 | 30 | 79 | NA |
| Optimised diet_best^¥^ | 76 | 192 | 77 | 121 | 64 | 100 | 96 | 314 | 100 | 30 | 88 | NA |
| Minimised^¶^ | 30 | 70 | 42 | 45 | 28 | 31 | 37 | 55 | 66 | 10 | 43 | 2 |
| Fruit 7 serves/w | 31 | 76 | 42 | 46 | 29 | 36 | 39 | 56 | 67 | 10 | 44 | 2 |
| Fruit 14 serves/w | 33 | 84 | 42 | 50 | 30 | 46 | 41 | 56 | 68 | 11 | 47 | 2 |
| Vitamin C-rich Fruit 5 serves/w | 32 | 126 | 44 | 47 | 29 | 34 | 43 | 56 | 69 | 10 | 44 | 2 |
| Vegetables 21 serves/w | 31 | 72 | 42 | 46 | 28 | 32 | 37 | 55 | 66 | 10 | 43 | 2 |
| Vegetables 28 serves/w | 31 | 74 | 42 | 46 | 29 | 32 | 38 | 56 | 67 | 10 | 44 | 2 |
| Green leafy vegetables 2 serves/w | 34 | 85 | 43 | 48 | 29 | 35 | 39 | 55 | 75 | 11 | 45 | 2 |
| Dairy 7 serves/w | 42 | 70 | 43 | 61 | 28 | 31 | 37 | 76 | 69 | 10 | 47 | 3 |
| Dairy 14 serves/w | 54 | 70 | 44 | 77 | 28 | 31 | 38 | 96 | 72 | 10 | 51 | 4 |
| Meat, fish, eggs 7 serves/w | 31 | 71 | 42 | 50 | 32 | 34 | 37 | 159 | 67 | 10 | 45 | 3 |
| Meat, fish, eggs 12 serves/w | 32 | 71 | 43 | 54 | 35 | 37 | 38 | 272 | 68 | 10 | 46 | 3 |
| Small fish with bones 4 serves/w | 31 | 70 | 42 | 48 | 31 | 33 | 37 | 133 | 67 | 10 | 44 | 3 |
| Small fish with bones 7 serves/w | 32 | 71 | 42 | 50 | 33 | 35 | 37 | 201 | 67 | 10 | 45 | 3 |
| Eggs 2 serves/w | 31 | 70 | 42 | 48 | 28 | 31 | 38 | 59 | 67 | 10 | 44 | 2 |
| Legumes 7 serves/w | 31 | 70 | 42 | 46 | 28 | 32 | 42 | 55 | 66 | 10 | 47 | 2 |
| Legumes 14 serves/w | 33 | 71 | 45 | 47 | 30 | 33 | 54 | 55 | 66 | 13 | 52 | 2 |
| Beans or lentils 4 serves/w | 32 | 71 | 43 | 46 | 29 | 32 | 46 | 55 | 66 | 13 | 47 | 2 |
| Nuts-seeds 7 serves/w | 31 | 70 | 45 | 46 | 33 | 32 | 43 | 55 | 66 | 10 | 47 | 2 |
| Roots 7 serves/w | 32 | 76 | 43 | 46 | 29 | 36 | 39 | 56 | 66 | 10 | 43 | 2 |
| Vitamin A-rich sweet potato 7 serves/w | 36 | 76 | 43 | 52 | 30 | 41 | 39 | 56 | 90 | 10 | 43 | 2 |
| Unrefined grain cereals 14 serves/w | 30 | 70 | 44 | 46 | 29 | 39 | 37 | 55 | 66 | 16 | 61 | 2 |
| Unrefined grain cereals 21 serves/w | 31 | 70 | 50 | 49 | 34 | 49 | 39 | 55 | 66 | 22 | 78 | 3 |
| Millet flour 4 serves/w | 30 | 70 | 46 | 53 | 34 | 32 | 42 | 55 | 66 | 11 | 47 | 2 |

† WHO recommended nutrient intakes for all nutrient except zinc (FAO/WHO/UNU, 2004; FAO/WHO, 2004). For zinc it was the iZinCg recommended nutrient intakes (Hotz & Brown, 2004). These results were used to select eight individual food-based recommendations to test in a systematically combined analysis in Module III.

^‡^ Abbreviations:^:^ Vit - vitamin

^§^ number of nutrients that were ≥65% in the Module III minimised diet for that nutrients

^¥^ Optimised diet_average - Module II optimised diet with both nutrient and food pattern goals; and Optimised diet_best - Module II optimised diet with only nutrient goals

^¶^ Minimised – the eleven Module III diets in which the objective function minimised the content of each nutrient; when a food-based recommendation was not tested

**Supplementary Material Table 5:** Module III minimisation results for the food-based recommendations tested compared expressed as a percentage of the recommended nutrient intakes and the number of nutrients that are ≥65% of the RNI for each set of FBRs tested

| **Analysis^‡^** | **Ca** | **Vit^‡^ C** | **B1** | **B2** | **B3** | **B6** | **Folate** | **B12** | **Vit A** | **Fe** | **Zn** | **>65** |
| --- | --- | --- | --- | --- | --- | --- | --- | --- | --- | --- | --- | --- |
|  | **%** | **%** | **%** | **%** | **%** | **%** | **%** | **%** | **%** | **%** | **%** | **% RNI^§^** |
| No Recommendations | 30.4 | 70.4 | 42 | 45.4 | 28.1 | 31.1 | 36.9 | 55.3 | 66.3 | 9.7 | 43.3 | 2 |
| Leg14 - Frt7 - MFE7 - SPot7 - Wgrt14 | 42.6 | 109.4 | 61.1 | 65.4 | 45.8 | 68.1 | 66.6 | 159.7 | 93.3 | 27.2 | 83.7 | 7 |
| Leg14 - Frt7 - MFE7 - Veg21 - SPot7 - WGrt14 | 43.5 | 137.5 | 61.6 | 66.9 | 46.8 | 69.3 | 71.4 | 159.8 | 95.3 | 28.9 | 84.8 | 7 |
| Dairy14 - Leg14 - Frt14 - SPot7 | 65.1 | 91 | 50.1 | 91.8 | 34.2 | 58.4 | 66.4 | 96.5 | 97.6 | 18.4 | 65 | 7 |
| Dairy14 - Leg14 - Frt14 - MFE7 - SPot7 | 66.1 | 91.1 | 51.1 | 96.4 | 37.6 | 61.7 | 66.7 | 200.1 | 98.2 | 19 | 67 | 7 |
| Dairy14 - Leg14 - Frt14 - Veg21 - SPot7 | 65.2 | 92.4 | 50.3 | 92 | 34.6 | 58.9 | 66.7 | 96.6 | 97.8 | 18.5 | 65.2 | 7 |
| Dairy14 - Leg14 - Frt14 - MFE7 - Veg21 - SPot7 | 66.2 | 92.6 | 51.4 | 96.6 | 38 | 62.3 | 67.1 | 200.2 | 98.4 | 19.1 | 67.2 | 7 |
| Dairy7 - Leg7 - Frt7 - MFE7 - SPot7 - WGrt14 | 52.2 | 110 | 60.9 | 80.4 | 43.7 | 66.8 | 56.6 | 179.8 | 96.1 | 23.3 | 82.9 | 6 |
| Dairy7 - Frt14 - SPot7 - WGrt14 | 50.9 | 90 | 55.5 | 77.9 | 37.7 | 65.7 | 47.7 | 76.5 | 94.8 | 21.9 | 76.9 | 6 |
| Frt14 - MFE7 - SPot7 - WGrt14 | 40.2 | 90.2 | 53.9 | 65.9 | 41.1 | 68.1 | 47 | 159.9 | 92.8 | 22.8 | 73.2 | 6 |
| Dairy7 - Frt14 - Veg21 - SPot7 - WGrt14 | 51 | 91.5 | 55.8 | 78.1 | 38.2 | 66.3 | 48.1 | 76.5 | 95.1 | 22 | 77.2 | 6 |
| Leg7 - Frt14 - MFE7 - SPot7 - WGrt14 | 41.4 | 100.4 | 58.8 | 67.1 | 43.7 | 71.1 | 56 | 159.9 | 92.8 | 24.3 | 78.3 | 6 |
| Frt14 - MFE7 - Veg21 - SPot7 - WGrt14 | 40.4 | 91.6 | 54.1 | 66.1 | 41.6 | 68.7 | 47.4 | 160 | 93 | 23 | 73.5 | 6 |
| Leg7 - Frt14 - MFE7 - Veg21 - SPot7 - WGrt14 | 41.7 | 105.9 | 59.3 | 67.3 | 44.6 | 71.7 | 56.8 | 160 | 93.3 | 24.5 | 78.5 | 6 |
| Dairy7 - Leg14 - Frt14 - WGrt14 | 47.5 | 85.5 | 54.9 | 71.4 | 37 | 57.2 | 65.5 | 75.9 | 70.7 | 24.9 | 81.7 | 6 |
| Dairy14 - Leg14 - Frt14 - WGrt14 | 59.3 | 85.5 | 58 | 88.3 | 37.7 | 59.3 | 66.9 | 96.1 | 73.4 | 24.9 | 88.6 | 6 |
| Dairy7 - Leg14 - Frt14 - MFE7 - WGrt14 | 48.6 | 85.6 | 56.4 | 76.4 | 41.1 | 61.7 | 66.1 | 179.5 | 71.3 | 25.9 | 84.9 | 6 |
| Dairy7 - Leg14 - Frt14 - Veg21 - WGrt14 | 47.7 | 86.9 | 55.2 | 71.6 | 37.4 | 57.8 | 65.9 | 76 | 70.9 | 25.1 | 82 | 6 |
| Dairy14 - Leg14 - Frt14 - MFE7 - WGrt14 | 60.8 | 99.4 | 61.4 | 93.3 | 42.1 | 63.9 | 68.9 | 199.7 | 74.1 | 25.9 | 91.8 | 6 |
| Dairy14 - Leg14 - Frt14 - Veg21 - WGrt14 | 59.4 | 86.9 | 58.2 | 88.5 | 38.2 | 59.9 | 67.3 | 96.1 | 73.6 | 25.1 | 88.9 | 6 |
| Dairy7 - Leg14 - Frt14 - MFE7 - Veg21 - WGrt14 | 48.7 | 87 | 56.6 | 76.5 | 41.6 | 62.3 | 66.5 | 179.6 | 71.5 | 26.1 | 85.2 | 6 |
| Dairy14 - Leg14 - Frt14 - MFE7 - Veg21 - WGrt14 | 61.1 | 104.9 | 61.9 | 93.6 | 42.9 | 64.5 | 69.7 | 199.8 | 74.6 | 26.1 | 92.1 | 6 |
| Dairy14 - WGrt14 | 53.7 | 70.4 | 48.8 | 79.1 | 29.4 | 41.7 | 39.2 | 95.6 | 71.7 | 16.2 | 72.9 | 5 |
| Dairy7 - MFE7 - WGrt14 | 43 | 70.5 | 47.5 | 67.4 | 33.2 | 44.4 | 38.6 | 179.1 | 69.7 | 16.9 | 69.6 | 5 |
| Dairy7 - SPot7 - WGrt14 | 48.2 | 75.9 | 51.9 | 71.6 | 34.1 | 56.5 | 42.3 | 76 | 93.1 | 20.1 | 73.9 | 5 |
| Dairy14 - Leg7 - WGrt14 | 54.4 | 70.4 | 50.1 | 79.7 | 30.3 | 43.8 | 44.7 | 95.6 | 71.7 | 17.3 | 77.3 | 5 |
| Dairy14 - Frt7 - WGrt14 | 54.4 | 76 | 49.8 | 80.6 | 30.6 | 44.5 | 41.3 | 95.8 | 72.3 | 16.3 | 73 | 5 |
| Dairy14 - MFE7 - WGrt14 | 54.7 | 70.5 | 49.9 | 83.8 | 33.2 | 46 | 39.6 | 199.2 | 72.4 | 16.9 | 75.6 | 5 |
| Dairy14 - Veg21 - WGrt14 | 53.8 | 71.8 | 49 | 79.3 | 29.9 | 42.2 | 39.5 | 95.7 | 71.9 | 16.3 | 73.2 | 5 |
| Dairy14 - SPot7 - WGrt14 | 59.9 | 75.9 | 55 | 88.4 | 34.9 | 58.6 | 43.7 | 96.1 | 95.9 | 20.2 | 80.8 | 5 |
| Dairy7 - Leg7 - MFE7 - WGrt14 | 43.8 | 70.5 | 48.8 | 68 | 34.1 | 46.4 | 44.1 | 179.1 | 69.7 | 18.1 | 74 | 5 |
| Dairy7 - Leg7 - SPot7 - WGrt14 | 48.9 | 75.9 | 53.8 | 72.6 | 35.6 | 58.9 | 48.1 | 76 | 93.1 | 21.6 | 78.9 | 5 |
| Dairy7 - Frt7 - MFE7 - WGrt14 | 43.8 | 76.1 | 48.5 | 68.9 | 34.4 | 47.2 | 40.6 | 179.3 | 70.3 | 17 | 69.7 | 5 |
| Dairy7 - Frt7 - SPot7 - WGrt14 | 48.9 | 81.5 | 53.5 | 73.4 | 35.8 | 59.5 | 44.7 | 76.2 | 93.7 | 20.5 | 74.5 | 5 |
| Dairy7 - MFE7 - Veg21 - WGrt14 | 43.2 | 71.9 | 47.7 | 67.5 | 33.6 | 44.9 | 39 | 179.1 | 69.9 | 17 | 69.9 | 5 |
| Dairy7 - MFE7 - SPot7 - WGrt14 | 49.2 | 76 | 53.4 | 76.5 | 38.3 | 61.1 | 42.9 | 179.6 | 93.8 | 21.1 | 77.1 | 5 |
| Dairy7 - Veg21 - SPot7 - WGrt14 | 48.3 | 77.3 | 52.1 | 71.8 | 34.6 | 57.1 | 42.7 | 76 | 93.3 | 20.3 | 74.2 | 5 |
| Dairy14 - Leg7 - Frt7 - WGrt14 | 55.2 | 76 | 51.2 | 81.3 | 31.6 | 46.8 | 46.9 | 95.8 | 72.3 | 17.5 | 77.4 | 5 |
| Dairy14 - Leg7 - MFE7 - WGrt14 | 55.5 | 70.5 | 51.2 | 84.4 | 34.2 | 48.2 | 45.2 | 199.2 | 72.4 | 18.1 | 80.1 | 5 |
| Dairy14 - Leg7 - Veg21 - WGrt14 | 54.5 | 71.8 | 50.3 | 79.9 | 30.8 | 44.4 | 45.1 | 95.7 | 71.9 | 17.4 | 77.6 | 5 |
| Dairy14 - Leg7 - SPot7 - WGrt14 | 60.7 | 75.9 | 57.3 | 89.6 | 36.3 | 61.1 | 50.2 | 96.1 | 95.9 | 21.6 | 85.8 | 5 |
| Dairy14 - Frt7 - MFE7 - WGrt14 | 55.5 | 76.1 | 50.9 | 85.3 | 34.4 | 48.9 | 41.7 | 199.4 | 73 | 17 | 75.7 | 5 |
| Dairy14 - Frt7 - Veg21 - WGrt14 | 54.5 | 77.4 | 50 | 80.8 | 31.1 | 45.1 | 41.6 | 95.9 | 72.5 | 16.4 | 73.2 | 5 |
| Dairy14 - Frt7 - SPot7 - WGrt14 | 60.7 | 81.5 | 57 | 90.3 | 36.5 | 61.7 | 46.1 | 96.4 | 96.5 | 20.6 | 81.4 | 5 |
| Dairy14 - MFE7 - Veg21 - WGrt14 | 54.9 | 71.9 | 50.1 | 84 | 33.7 | 46.6 | 40 | 199.2 | 72.6 | 17 | 75.9 | 5 |
| Dairy14 - MFE7 - SPot7 - WGrt14 | 61 | 76 | 56.4 | 93.4 | 39 | 63.2 | 44.3 | 199.7 | 96.5 | 21.1 | 84 | 5 |
| Dairy14 - Veg21 - SPot7 - WGrt14 | 60 | 77.3 | 55.2 | 88.6 | 35.3 | 59.2 | 44.1 | 96.2 | 96.1 | 20.3 | 81.1 | 5 |
| Dairy7 - Leg7 - Frt7 - MFE7 - WGrt14 | 44.5 | 76.1 | 49.8 | 69.5 | 35.3 | 49.4 | 46.2 | 179.3 | 70.3 | 18.4 | 74.1 | 5 |
| Dairy7 - Leg7 - Frt7 - Veg21 - WGrt14 | 43.6 | 77.4 | 48.9 | 65 | 31.9 | 45.8 | 46.1 | 75.7 | 69.8 | 17.6 | 71.6 | 5 |
| Dairy7 - Leg7 - Frt7 - SPot7 - WGrt14 | 49.7 | 81.5 | 55.5 | 74.4 | 37.3 | 62 | 50.5 | 76.2 | 93.7 | 22 | 79.5 | 5 |
| Dairy7 - Leg7 - MFE7 - Veg21 - WGrt14 | 43.9 | 71.9 | 49 | 68.1 | 34.5 | 47 | 44.5 | 179.1 | 69.9 | 18.2 | 74.2 | 5 |
| Dairy7 - Leg7 - MFE7 - SPot7 - WGrt14 | 50 | 76 | 55.3 | 77.5 | 39.7 | 63.5 | 48.8 | 179.6 | 93.8 | 22.6 | 82.1 | 5 |
| Dairy7 - Leg7 - Veg21 - SPot7 - WGrt14 | 49.1 | 77.3 | 54.1 | 72.8 | 36.1 | 59.5 | 48.5 | 76 | 93.3 | 21.8 | 79.2 | 5 |
| Dairy7 - Frt7 - MFE7 - Veg21 - WGrt14 | 43.9 | 77.5 | 48.7 | 69 | 34.8 | 47.7 | 41 | 179.3 | 70.5 | 17.2 | 69.9 | 5 |
| Dairy7 - Frt7 - MFE7 - SPot7 - WGrt14 | 50 | 81.6 | 55 | 78.3 | 39.9 | 64.1 | 45.3 | 179.8 | 94.4 | 21.5 | 77.7 | 5 |
| Dairy7 - Frt7 - Veg21 - SPot7 - WGrt14 | 49 | 82.9 | 53.7 | 73.6 | 36.3 | 60.1 | 45.1 | 76.2 | 93.9 | 20.7 | 74.8 | 5 |
| Dairy7 - MFE7 - Veg21 - SPot7 - WGrt14 | 49.4 | 77.4 | 53.6 | 76.7 | 38.7 | 61.7 | 43.3 | 179.6 | 94 | 21.3 | 77.4 | 5 |
| Dairy14 - Leg7 - Frt7 - MFE7 - WGrt14 | 56.2 | 76.1 | 52.6 | 86.2 | 35.6 | 51.2 | 47.5 | 199.4 | 73 | 18.4 | 80.5 | 5 |
| Dairy14 - Leg7 - Frt7 - Veg21 - WGrt14 | 55.3 | 77.4 | 51.4 | 81.4 | 32 | 47.4 | 47.3 | 95.9 | 72.5 | 17.6 | 77.7 | 5 |
| Dairy14 - Leg7 - MFE7 - Veg21 - WGrt14 | 55.6 | 71.9 | 51.4 | 84.6 | 34.6 | 48.8 | 45.6 | 199.2 | 72.6 | 18.2 | 80.3 | 5 |
| Dairy14 - Leg7 - Veg21 - SPot7 - WGrt14 | 60.8 | 77.3 | 57.6 | 89.8 | 36.9 | 61.7 | 51.2 | 96.2 | 96.2 | 21.8 | 86.1 | 5 |
| Dairy14 - Frt7 - MFE7 - Veg21 - WGrt14 | 55.6 | 77.6 | 51.1 | 85.5 | 34.9 | 49.5 | 42.1 | 199.4 | 73.2 | 17.2 | 76 | 5 |
| Dairy14 - Frt7 - Veg21 - SPot7 - WGrt14 | 60.9 | 86 | 57.5 | 90.5 | 37 | 62.3 | 46.5 | 96.4 | 96.7 | 20.7 | 81.7 | 5 |
| Dairy14 - MFE7 - Veg21 - SPot7 - WGrt14 | 61.1 | 77.4 | 56.7 | 93.6 | 39.5 | 63.8 | 44.7 | 199.8 | 96.8 | 21.3 | 84.3 | 5 |
| Dairy7 - Leg7 - Frt7 - MFE7 - Veg21 - WGrt14 | 44.6 | 77.5 | 50 | 69.6 | 35.8 | 50 | 46.6 | 179.3 | 70.5 | 18.5 | 74.3 | 5 |
| Dairy7 - Leg7 - Frt7 - Veg21 - SPot7 - WGrt14 | 49.8 | 82.9 | 55.8 | 74.6 | 37.7 | 62.5 | 50.9 | 76.3 | 93.9 | 22.2 | 79.8 | 5 |
| Dairy7 - Leg7 - MFE7 - Veg21 - SPot7 - WGrt14 | 50.1 | 77.4 | 55.5 | 77.7 | 40.2 | 64.1 | 49.2 | 179.6 | 94 | 22.7 | 82.4 | 5 |
| Dairy7 - Frt7 - MFE7 - Veg21 - SPot7 - WGrt14 | 50.1 | 83.1 | 55.2 | 78.5 | 40.4 | 64.7 | 45.7 | 179.8 | 94.6 | 21.7 | 78 | 5 |
| Dairy14 - Leg7 - Frt7 - MFE7 - Veg21 - WGrt14 | 56.4 | 77.6 | 52.8 | 86.3 | 36.1 | 51.8 | 47.9 | 199.4 | 73.2 | 18.5 | 80.8 | 5 |
| Leg7 - Frt7 - MFE7 - Veg21 - SPot7 - WGrt14 | 39.2 | 83 | 54.1 | 62.6 | 41.1 | 65 | 50.2 | 159.7 | 91.9 | 23.1 | 76.1 | 5 |
| Dairy7 - Frt14 - WGrt14 | 44.6 | 84.5 | 48.7 | 68.3 | 32.1 | 49.9 | 43 | 75.9 | 70.7 | 17.3 | 68.8 | 5 |
| Dairy14 - Frt14 - WGrt14 | 56.3 | 84.5 | 51.2 | 84.7 | 32.1 | 50.9 | 44.1 | 96.1 | 73.4 | 17.3 | 74.8 | 5 |
| Dairy7 - Leg7 - Frt14 - WGrt14 | 45.4 | 84.5 | 50.6 | 69 | 33.5 | 52 | 48.8 | 75.9 | 70.7 | 18.7 | 73.3 | 5 |
| Dairy7 - Frt14 - MFE7 - WGrt14 | 45.7 | 84.7 | 49.8 | 72.9 | 35.9 | 53.8 | 43.4 | 179.5 | 71.3 | 18.2 | 71.5 | 5 |
| Dairy7 - Frt14 - Veg21 - WGrt14 | 44.8 | 85.9 | 48.9 | 68.5 | 32.5 | 50.5 | 43.3 | 76 | 70.9 | 17.4 | 69 | 5 |
| Dairy14 - Leg7 - Frt14 - WGrt14 | 57.1 | 84.5 | 53.5 | 85.7 | 34 | 53.3 | 50.1 | 96.1 | 73.4 | 18.7 | 79.7 | 5 |
| Dairy14 - Frt14 - MFE7 - WGrt14 | 57.4 | 84.7 | 52.6 | 89.6 | 36.1 | 54.9 | 44.7 | 199.6 | 74.1 | 18.2 | 77.9 | 5 |
| Dairy14 - Frt14 - Veg21 - WGrt14 | 56.5 | 86 | 51.4 | 84.9 | 32.6 | 51.4 | 44.5 | 96.1 | 73.6 | 17.4 | 75.1 | 5 |
| Dairy7 - Leg7 - Frt14 - MFE7 - WGrt14 | 46.5 | 84.7 | 51.9 | 73.8 | 37.4 | 55.9 | 49.3 | 179.5 | 71.3 | 19.7 | 76.1 | 5 |
| Dairy7 - Leg7 - Frt14 - Veg21 - WGrt14 | 45.5 | 85.9 | 50.8 | 69.2 | 34 | 52.5 | 49.1 | 76 | 70.9 | 18.8 | 73.5 | 5 |
| Dairy7 - Frt14 - MFE7 - Veg21 - WGrt14 | 45.8 | 86.1 | 50 | 73.1 | 36.3 | 54.4 | 43.8 | 179.6 | 71.5 | 18.3 | 71.7 | 5 |
| Dairy14 - Leg7 - Frt14 - MFE7 - WGrt14 | 58.2 | 84.7 | 55 | 90.6 | 38.1 | 57.8 | 50.7 | 199.6 | 74.1 | 19.7 | 82.9 | 5 |
| Dairy14 - Leg7 - Frt14 - Veg21 - WGrt14 | 57.2 | 86 | 53.8 | 85.9 | 34.5 | 53.9 | 50.5 | 96.1 | 73.6 | 18.8 | 80 | 5 |
| Dairy14 - Frt14 - MFE7 - Veg21 - WGrt14 | 57.5 | 86.1 | 52.9 | 89.8 | 36.5 | 55.5 | 45.1 | 199.7 | 74.3 | 18.3 | 78.2 | 5 |
| Dairy7 - Leg7 - Frt14 - MFE7 - Veg21 - WGrt14 | 46.6 | 86.1 | 52.2 | 74 | 37.9 | 56.4 | 49.7 | 179.6 | 71.5 | 19.8 | 76.4 | 5 |
| Dairy14 - Leg7 - Frt14 - MFE7 - Veg21 - WGrt14 | 58.3 | 86.1 | 55.2 | 90.8 | 38.6 | 58.4 | 51.1 | 199.7 | 74.3 | 19.8 | 83.2 | 5 |
| Dairy14 - Leg14 - WGrt14 | 56.3 | 71 | 53.9 | 81.4 | 32.8 | 46.8 | 57.6 | 95.6 | 71.7 | 21.1 | 84.3 | 5 |
| Dairy7 - Leg14 - Frt7 - WGrt14 | 45.3 | 76.6 | 52.4 | 66.4 | 33.7 | 48.1 | 58.7 | 75.7 | 69.6 | 21.5 | 78.1 | 5 |
| Dairy7 - Leg14 - MFE7 - WGrt14 | 45.7 | 71.1 | 52.4 | 69.5 | 36.3 | 49.3 | 56.9 | 179.1 | 69.7 | 22.1 | 80.8 | 5 |
| Dairy7 - Leg14 - Veg21 - WGrt14 | 44.7 | 72.4 | 51.5 | 65.1 | 33 | 45.5 | 56.8 | 75.5 | 69.2 | 21.3 | 78.2 | 5 |
| Dairy7 - Leg14 - SPot7 - WGrt14 | 50.8 | 76.5 | 58.4 | 74.6 | 38.5 | 62.2 | 61.2 | 76 | 93.1 | 25.7 | 86.5 | 5 |
| Dairy14 - Leg14 - Frt7 - WGrt14 | 57 | 76.6 | 55.5 | 83.2 | 34.4 | 49.9 | 60 | 95.8 | 72.3 | 21.5 | 84.9 | 5 |
| Dairy14 - Leg14 - MFE7 - WGrt14 | 57.4 | 71.1 | 55.3 | 86.3 | 36.9 | 51.4 | 58.2 | 199.2 | 72.4 | 22.1 | 87.5 | 5 |
| Dairy14 - Leg14 - Veg21 - WGrt14 | 56.4 | 72.4 | 54.1 | 81.6 | 33.2 | 47.4 | 58 | 95.7 | 71.9 | 21.3 | 84.6 | 5 |
| Dairy7 - Leg14 - Frt7 - MFE7 - WGrt14 | 46.4 | 76.7 | 53.9 | 71.2 | 37.8 | 52.3 | 59.2 | 179.3 | 70.3 | 22.5 | 81.2 | 5 |
| Dairy7 - Leg14 - Frt7 - Veg21 - WGrt14 | 45.5 | 78 | 52.7 | 66.6 | 34.2 | 48.6 | 59 | 75.7 | 69.8 | 21.6 | 78.4 | 5 |
| Dairy7 - Leg14 - MFE7 - Veg21 - WGrt14 | 45.8 | 72.5 | 52.6 | 69.7 | 36.8 | 49.9 | 57.3 | 179.1 | 69.9 | 22.2 | 81 | 5 |
| Dairy7 - Leg14 - Veg21 - SPot7 - WGrt14 | 51 | 77.9 | 58.8 | 74.8 | 39.6 | 62.8 | 61.6 | 76 | 93.4 | 25.8 | 86.8 | 5 |
| Dairy14 - Leg14 - Frt7 - MFE7 - WGrt14 | 58.1 | 76.7 | 56.9 | 88.1 | 38.5 | 54.4 | 60.6 | 199.4 | 73 | 22.5 | 88.1 | 5 |
| Dairy14 - Leg14 - Frt7 - Veg21 - WGrt14 | 57.2 | 78 | 55.7 | 83.4 | 34.9 | 50.4 | 60.4 | 95.9 | 72.5 | 21.6 | 85.2 | 5 |
| Dairy14 - Leg14 - MFE7 - Veg21 - WGrt14 | 57.5 | 72.5 | 55.6 | 86.5 | 37.3 | 52 | 58.6 | 199.2 | 72.6 | 22.2 | 87.8 | 5 |
| Leg14 - MFE7 - Veg21 - SPot7 - WGrt14 | 40.3 | 78 | 56.8 | 62.8 | 42.4 | 65.2 | 60.9 | 159.5 | 91.3 | 26.8 | 83.1 | 5 |
| Dairy7 - Leg14 - Frt7 - MFE7 - Veg21 - WGrt14 | 46.5 | 78.1 | 54.1 | 71.4 | 38.3 | 52.9 | 59.6 | 179.3 | 70.5 | 22.6 | 81.5 | 5 |
| Dairy14 - Leg14 - Frt7 - MFE7 - Veg21 - WGrt14 | 58.3 | 78.1 | 57.2 | 88.3 | 39 | 55 | 61 | 199.5 | 73.2 | 22.6 | 88.4 | 5 |
| Dairy7 - Leg14 - Frt14 - SPot7 | 53.4 | 91 | 48.3 | 75.6 | 34.2 | 58.4 | 65.7 | 76.4 | 94.8 | 18.4 | 60.6 | 5 |
| Dairy14 - Leg14 - Frt14 - MFE7 | 60.1 | 85.6 | 48.6 | 88.6 | 36 | 52.2 | 63.9 | 199.6 | 74.1 | 17.7 | 66 | 5 |
| Dairy7 - Leg14 - Frt14 - MFE7 - SPot7 | 54.5 | 91.1 | 48.8 | 80.1 | 37.6 | 61.7 | 65.9 | 180 | 95.5 | 19 | 62.3 | 5 |
| Dairy7 - Leg14 - Frt14 - Veg21 - SPot7 | 53.5 | 92.4 | 48.5 | 75.8 | 34.6 | 58.9 | 66.1 | 76.5 | 95 | 18.5 | 60.7 | 5 |
| Dairy14 - Leg14 - Frt14 - MFE7 - Veg21 | 60.2 | 87 | 48.7 | 88.7 | 36.4 | 52.7 | 64.3 | 199.7 | 74.3 | 17.8 | 66.1 | 5 |
| Leg14 - Frt14 - MFE7 - Veg21 - WGrt14 | 37 | 87 | 53.6 | 59.7 | 40.8 | 60.3 | 65.1 | 159.4 | 68.8 | 26.1 | 78.3 | 5 |
| Dairy7 - Leg14 - Frt14 - MFE7 - Veg21 - SPot7 | 54.6 | 92.5 | 49 | 80.3 | 38 | 62.2 | 66.2 | 180 | 95.7 | 19.1 | 62.5 | 5 |
| Dairy14 | 53.7 | 70.4 | 43.8 | 77.1 | 28.1 | 31.1 | 38 | 95.6 | 71.7 | 9.7 | 51 | 4 |
| Dairy7 - MFE7 | 43 | 70.5 | 43.2 | 65.6 | 31.5 | 34 | 37.6 | 179.1 | 69.7 | 9.8 | 48.6 | 4 |
| Dairy7 - SPot7 | 48 | 75.9 | 43.7 | 68.2 | 29.6 | 40.8 | 39.9 | 75.9 | 93.1 | 10.4 | 47.1 | 4 |
| Dairy7 - WGrt14 | 42 | 70.4 | 46.4 | 62.7 | 29.4 | 40.1 | 38.3 | 75.5 | 69 | 16.2 | 67 | 4 |
| Dairy14 - Leg7 | 54.4 | 70.4 | 44.3 | 77.3 | 28.3 | 31.7 | 43.1 | 95.6 | 71.7 | 10.2 | 54.4 | 4 |
| Dairy14 - Frt7 | 54.3 | 76 | 43.8 | 78.2 | 28.6 | 36 | 39.7 | 95.8 | 72.3 | 9.8 | 51.3 | 4 |
| Dairy14 - MFE7 | 54.7 | 70.5 | 44.1 | 81.4 | 31.5 | 34 | 38.1 | 199.2 | 72.4 | 9.8 | 52.5 | 4 |
| Dairy14 - Veg21 | 53.8 | 71.8 | 44 | 77.2 | 28.5 | 31.6 | 38.3 | 95.7 | 71.9 | 9.8 | 51.1 | 4 |
| Dairy14 - SPot7 | 59.7 | 75.9 | 44.6 | 84 | 29.6 | 40.8 | 40.4 | 96.1 | 95.9 | 10.4 | 51.3 | 4 |
| Dairy7 - Leg7 - MFE7 | 43.8 | 70.5 | 43.7 | 65.8 | 31.7 | 34.8 | 42.7 | 179.1 | 69.7 | 10.3 | 52 | 4 |
| Dairy7 - Leg7 - SPot7 | 48.8 | 75.9 | 44.2 | 68.4 | 29.8 | 42.2 | 45 | 75.9 | 93.1 | 11.4 | 50.5 | 4 |
| Dairy7 - Leg7 - WGrt14 | 42.7 | 70.4 | 47.7 | 63.3 | 30.3 | 42.1 | 43.7 | 75.5 | 69 | 17.3 | 71.3 | 4 |
| Dairy7 - Frt7 - MFE7 | 43.7 | 76.1 | 43.2 | 66.7 | 32 | 38.9 | 39.4 | 179.2 | 70.3 | 9.9 | 48.9 | 4 |
| Dairy7 - Frt7 - SPot7 | 48.7 | 81.5 | 43.7 | 69.2 | 30.1 | 45 | 41.7 | 76.1 | 93.7 | 10.5 | 47.4 | 4 |
| Dairy7 - Frt7 - WGrt14 | 42.7 | 76 | 47.4 | 64.2 | 30.6 | 43.2 | 40.3 | 75.7 | 69.6 | 16.3 | 67 | 4 |
| Dairy7 - MFE7 - Veg21 | 43.2 | 71.9 | 43.3 | 65.7 | 31.9 | 34.5 | 37.9 | 179.1 | 69.9 | 9.9 | 48.8 | 4 |
| Dairy7 - MFE7 - SPot7 | 49.1 | 76 | 44 | 72.5 | 33 | 44.2 | 40 | 179.5 | 93.8 | 11 | 48.7 | 4 |
| Dairy7 - Veg21 - SPot7 | 48.2 | 77.3 | 43.9 | 68.3 | 30 | 41.3 | 40.2 | 76 | 93.3 | 10.5 | 47.2 | 4 |
| Dairy7 - Veg21 - WGrt14 | 42.1 | 71.8 | 46.6 | 62.9 | 29.8 | 40.7 | 38.7 | 75.5 | 69.2 | 16.3 | 67.2 | 4 |
| Dairy14 - Leg7 - Frt7 | 55.1 | 76 | 44.3 | 78.4 | 28.8 | 36.6 | 45 | 95.8 | 72.3 | 10.3 | 54.7 | 4 |
| Dairy14 - Leg7 - MFE7 | 55.4 | 70.5 | 44.6 | 81.6 | 31.7 | 34.8 | 43.2 | 199.2 | 72.4 | 10.3 | 55.9 | 4 |
| Dairy14 - Leg7 - Veg21 | 54.5 | 71.8 | 44.5 | 77.4 | 28.7 | 32.2 | 43.4 | 95.7 | 71.9 | 10.3 | 54.5 | 4 |
| Dairy14 - Leg7 - SPot7 | 60.4 | 75.9 | 45.1 | 84.2 | 29.8 | 42.4 | 45.5 | 96.1 | 95.9 | 11.4 | 55 | 4 |
| Dairy14 - Frt7 - MFE7 | 55.4 | 76.1 | 44.1 | 82.5 | 32 | 38.9 | 39.9 | 199.4 | 73 | 9.9 | 52.8 | 4 |
| Dairy14 - Frt7 - Veg21 | 54.5 | 77.4 | 44 | 78.3 | 29 | 36.5 | 40.1 | 95.9 | 72.5 | 9.8 | 51.4 | 4 |
| Dairy14 - Frt7 - SPot7 | 60.4 | 81.5 | 44.6 | 85.2 | 30.1 | 45 | 42.2 | 96.3 | 96.5 | 10.5 | 51.4 | 4 |
| Dairy14 - MFE7 - Veg21 | 54.8 | 71.9 | 44.2 | 81.6 | 31.9 | 34.5 | 38.5 | 199.2 | 72.6 | 9.9 | 52.7 | 4 |
| Dairy14 - MFE7 - SPot7 | 60.7 | 76 | 44.9 | 88.4 | 33 | 44.4 | 40.5 | 199.7 | 96.5 | 11 | 53.3 | 4 |
| Dairy14 - Veg21 - SPot7 | 59.8 | 77.3 | 44.8 | 84.2 | 30 | 41.4 | 40.8 | 96.1 | 96.1 | 10.5 | 51.5 | 4 |
| Leg7 - MFE7 - WGrt14 | 32.1 | 70.5 | 46.4 | 51.6 | 34 | 44.9 | 43.2 | 158.9 | 66.9 | 18.1 | 68 | 4 |
| MFE7 - SPot7 - WGrt14 | 37.5 | 76 | 50.4 | 59.6 | 37.6 | 59 | 41.6 | 159.4 | 91.1 | 21.1 | 70.2 | 4 |
| Dairy7 - Leg7 - Frt7 - MFE7 | 44.4 | 76.1 | 43.7 | 66.8 | 32.2 | 39.4 | 44.6 | 179.2 | 70.3 | 10.4 | 52.3 | 4 |
| Dairy7 - Leg7 - Frt7 - SPot7 | 49.4 | 81.5 | 44.2 | 69.4 | 30.3 | 46.3 | 46.9 | 76.1 | 93.7 | 11.4 | 50.8 | 4 |
| Dairy7 - Leg7 - Frt7 - WGrt14 | 43.5 | 76 | 48.7 | 64.8 | 31.5 | 45.2 | 45.8 | 75.7 | 69.6 | 17.5 | 71.3 | 4 |
| Dairy7 - Leg7 - MFE7 - Veg21 | 43.9 | 71.9 | 43.8 | 65.9 | 32.1 | 35.3 | 43 | 179.1 | 69.9 | 10.4 | 52.1 | 4 |
| Dairy7 - Leg7 - MFE7 - SPot7 | 49.8 | 76 | 44.5 | 72.7 | 33.3 | 45.6 | 45.1 | 179.5 | 93.8 | 12 | 52.3 | 4 |
| Dairy7 - Leg7 - Veg21 - SPot7 | 48.9 | 77.3 | 44.4 | 68.5 | 30.2 | 42.7 | 45.3 | 76 | 93.3 | 11.5 | 50.7 | 4 |
| Dairy7 - Leg7 - Veg21 - WGrt14 | 42.8 | 71.8 | 47.9 | 63.5 | 30.7 | 42.6 | 44.1 | 75.5 | 69.2 | 17.4 | 71.6 | 4 |
| Dairy7 - Frt7 - MFE7 - Veg21 | 43.9 | 77.5 | 43.3 | 66.8 | 32.4 | 39.4 | 39.7 | 179.3 | 70.5 | 9.9 | 49.1 | 4 |
| Dairy7 - Frt7 - MFE7 - SPot7 | 49.8 | 81.6 | 44 | 73.6 | 33.5 | 48.4 | 41.8 | 179.7 | 94.4 | 11 | 48.9 | 4 |
| Dairy7 - Frt7 - Veg21 - SPot7 | 48.8 | 82.9 | 43.9 | 69.4 | 30.5 | 45.5 | 42 | 76.2 | 93.9 | 10.6 | 47.5 | 4 |
| Dairy7 - Frt7 - Veg21 - WGrt14 | 42.8 | 77.4 | 47.6 | 64.4 | 31 | 43.8 | 40.6 | 75.7 | 69.8 | 16.4 | 67.2 | 4 |
| Dairy7 - MFE7 - Veg21 - SPot7 | 49.2 | 77.4 | 44.1 | 72.6 | 33.4 | 44.7 | 40.4 | 179.6 | 94 | 11.1 | 48.9 | 4 |
| Dairy14 - Leg7 - Frt7 - MFE7 | 56.1 | 76.1 | 44.6 | 82.7 | 32.2 | 39.4 | 45.2 | 199.4 | 73 | 10.4 | 56.2 | 4 |
| Dairy14 - Leg7 - Frt7 - Veg21 | 55.2 | 77.4 | 44.5 | 78.5 | 29.2 | 37 | 45.4 | 95.9 | 72.5 | 10.4 | 54.8 | 4 |
| Dairy14 - Leg7 - Frt7 - SPot7 | 61.1 | 81.5 | 45.1 | 85.5 | 30.3 | 46.4 | 47.5 | 96.3 | 96.5 | 11.4 | 55.1 | 4 |
| Dairy14 - Leg7 - MFE7 - Veg21 | 55.5 | 71.9 | 44.7 | 81.8 | 32.1 | 35.3 | 43.6 | 199.2 | 72.6 | 10.4 | 56 | 4 |
| Dairy14 - Leg7 - MFE7 - SPot7 | 61.4 | 76 | 45.4 | 88.7 | 33.3 | 46.2 | 45.7 | 199.7 | 96.5 | 12 | 57 | 4 |
| Dairy14 - Leg7 - Veg21 - SPot7 | 60.5 | 77.3 | 45.3 | 84.4 | 30.2 | 43 | 45.9 | 96.1 | 96.1 | 11.5 | 55.2 | 4 |
| Dairy14 - Frt7 - MFE7 - Veg21 | 55.5 | 77.6 | 44.2 | 82.7 | 32.4 | 39.4 | 40.2 | 199.4 | 73.2 | 9.9 | 52.9 | 4 |
| Dairy14 - Frt7 - MFE7 - SPot7 | 61.4 | 81.7 | 44.9 | 89.7 | 33.5 | 48.4 | 42.3 | 199.9 | 97.1 | 11 | 53.3 | 4 |
| Dairy14 - Frt7 - Veg21 - SPot7 | 60.5 | 82.9 | 44.8 | 85.3 | 30.5 | 45.5 | 42.5 | 96.3 | 96.7 | 10.6 | 51.6 | 4 |
| Dairy14 - MFE7 - Veg21 - SPot7 | 60.8 | 77.4 | 45 | 88.5 | 33.4 | 44.9 | 40.9 | 199.7 | 96.7 | 11.1 | 53.5 | 4 |
| Leg7 - Frt7 - MFE7 - WGrt14 | 32.8 | 76.1 | 47.4 | 53 | 35.3 | 48.2 | 45.1 | 159.1 | 67.5 | 18.4 | 68.1 | 4 |
| Leg7 - MFE7 - Veg21 - WGrt14 | 32.2 | 71.9 | 46.6 | 51.7 | 34.5 | 45.4 | 43.5 | 159 | 67.1 | 18.2 | 68.3 | 4 |
| Leg7 - MFE7 - SPot7 - WGrt14 | 38.3 | 76 | 52.2 | 60.6 | 39 | 61.4 | 47.4 | 159.4 | 91.1 | 22.6 | 75.2 | 4 |
| Frt7 - MFE7 - SPot7 - WGrt14 | 38.3 | 81.6 | 51.9 | 61.4 | 39.2 | 62 | 43.9 | 159.6 | 91.7 | 21.5 | 70.8 | 4 |
| MFE7 - Veg21 - SPot7 - WGrt14 | 37.7 | 77.4 | 50.6 | 59.8 | 38 | 59.6 | 42 | 159.5 | 91.3 | 21.3 | 70.5 | 4 |
| Dairy7 - Leg7 - Frt7 - MFE7 - Veg21 | 44.6 | 77.5 | 43.8 | 67 | 32.6 | 39.9 | 45 | 179.3 | 70.5 | 10.5 | 52.4 | 4 |
| Dairy7 - Leg7 - Frt7 - MFE7 - SPot7 | 50.5 | 81.6 | 44.5 | 73.9 | 33.8 | 49.7 | 47.1 | 179.7 | 94.4 | 12 | 52.4 | 4 |
| Dairy7 - Leg7 - Frt7 - Veg21 - SPot7 | 49.6 | 82.9 | 44.4 | 69.6 | 30.7 | 46.9 | 47.3 | 76.2 | 93.9 | 11.5 | 50.9 | 4 |
| Dairy7 - Leg7 - MFE7 - Veg21 - SPot7 | 49.9 | 77.4 | 44.6 | 72.8 | 33.7 | 46.2 | 45.5 | 179.6 | 94 | 12.1 | 52.5 | 4 |
| Dairy7 - Frt7 - MFE7 - Veg21 - SPot7 | 49.9 | 83.1 | 44.1 | 73.7 | 33.9 | 48.9 | 42.2 | 179.8 | 94.6 | 11.2 | 49.1 | 4 |
| Dairy14 - Leg7 - Frt7 - MFE7 - Veg21 | 56.2 | 77.6 | 44.7 | 82.9 | 32.6 | 39.9 | 45.5 | 199.4 | 73.2 | 10.5 | 56.3 | 4 |
| Dairy14 - Leg7 - Frt7 - MFE7 - SPot7 | 62.1 | 81.7 | 45.6 | 90.1 | 33.8 | 49.9 | 47.6 | 199.9 | 97.1 | 12 | 57 | 4 |
| Dairy14 - Leg7 - Frt7 - Veg21 - SPot7 | 61.2 | 82.9 | 45.3 | 85.7 | 30.7 | 46.9 | 47.8 | 96.3 | 96.7 | 11.5 | 55.3 | 4 |
| Dairy14 - Leg7 - MFE7 - Veg21 - SPot7 | 61.6 | 77.4 | 45.6 | 88.9 | 33.7 | 46.8 | 46.1 | 199.7 | 96.7 | 12.1 | 57.2 | 4 |
| Dairy14 - Frt7 - MFE7 - Veg21 - SPot7 | 61.5 | 83.1 | 45 | 89.8 | 33.9 | 48.9 | 42.7 | 199.9 | 97.3 | 11.2 | 53.5 | 4 |
| Leg7 - Frt7 - MFE7 - Veg21 - WGrt14 | 32.9 | 77.5 | 47.6 | 53.2 | 35.7 | 48.7 | 45.5 | 159.2 | 67.7 | 18.5 | 68.3 | 4 |
| Leg7 - Frt7 - MFE7 - SPot7 - WGrt14 | 39.1 | 81.6 | 53.8 | 62.4 | 40.7 | 64.4 | 49.8 | 159.6 | 91.7 | 23 | 75.8 | 4 |
| Leg7 - MFE7 - Veg21 - SPot7 - WGrt14 | 38.4 | 77.4 | 52.5 | 60.8 | 39.5 | 61.9 | 47.8 | 159.5 | 91.3 | 22.7 | 75.5 | 4 |
| Frt7 - MFE7 - Veg21 - SPot7 - WGrt14 | 38.4 | 83 | 52.1 | 61.6 | 39.7 | 62.6 | 44.3 | 159.7 | 91.9 | 21.7 | 71.1 | 4 |
| Dairy7 - Leg7 - Frt7 - MFE7 - Veg21 - SPot7 | 50.6 | 83.1 | 44.6 | 74.1 | 34.2 | 50.2 | 47.4 | 179.8 | 94.6 | 12.1 | 52.6 | 4 |
| Dairy14 - Leg7 - Frt7 - MFE7 - Veg21 - SPot7 | 62.2 | 83.1 | 45.8 | 90.3 | 34.2 | 50.4 | 48 | 199.9 | 97.3 | 12.1 | 57.3 | 4 |
| Dairy7 - Frt14 | 44.6 | 84.5 | 43.3 | 66.2 | 29.5 | 45.7 | 41.9 | 75.9 | 70.7 | 11.3 | 50.5 | 4 |
| Dairy14 - Frt14 | 56.2 | 84.5 | 44.2 | 82 | 29.5 | 45.7 | 42.4 | 96 | 73.4 | 11.3 | 54.3 | 4 |
| Dairy7 - Leg7 - Frt14 | 45.3 | 84.5 | 44.5 | 66.6 | 30.2 | 46.2 | 47.3 | 75.9 | 70.7 | 12.2 | 54.1 | 4 |
| Dairy7 - Frt14 - MFE7 | 45.6 | 84.7 | 43.6 | 70.5 | 32.9 | 48.6 | 42 | 179.5 | 71.3 | 11.4 | 51.9 | 4 |
| Dairy7 - Frt14 - Veg21 | 44.7 | 85.9 | 43.5 | 66.3 | 29.9 | 46.2 | 42.2 | 76 | 70.9 | 11.3 | 50.7 | 4 |
| Dairy7 - Frt14 - SPot7 | 50.6 | 90 | 44.1 | 73.1 | 31 | 54 | 44.3 | 76.4 | 94.8 | 11.4 | 50.5 | 4 |
| Dairy14 - Leg7 - Frt14 | 56.9 | 84.5 | 45.4 | 82.5 | 30.2 | 46.2 | 47.8 | 96 | 73.4 | 12.2 | 57.9 | 4 |
| Dairy14 - Frt14 - MFE7 | 57.3 | 84.7 | 44.5 | 86.4 | 32.9 | 48.6 | 42.5 | 199.6 | 74.1 | 11.4 | 55.8 | 4 |
| Dairy14 - Frt14 - Veg21 | 56.3 | 86 | 44.4 | 82.2 | 29.9 | 46.2 | 42.8 | 96.1 | 73.6 | 11.3 | 54.4 | 4 |
| Dairy14 - Frt14 - SPot7 | 62.3 | 90.1 | 45 | 89.1 | 31 | 54 | 44.8 | 96.5 | 97.6 | 11.4 | 54.3 | 4 |
| Frt14 - MFE7 - WGrt14 | 34 | 84.6 | 47.4 | 56.5 | 35.8 | 52.9 | 42.3 | 159.4 | 68.6 | 18.2 | 65.8 | 4 |
| Dairy7 - Leg7 - Frt14 - MFE7 | 46.3 | 84.7 | 44.7 | 70.9 | 33.7 | 49.1 | 47.4 | 179.5 | 71.3 | 12.3 | 55.6 | 4 |
| Dairy7 - Leg7 - Frt14 - Veg21 | 45.4 | 85.9 | 44.6 | 66.8 | 30.6 | 46.7 | 47.6 | 76 | 70.9 | 12.3 | 54.2 | 4 |
| Dairy7 - Leg7 - Frt14 - SPot7 | 51.3 | 90 | 45.3 | 73.5 | 31.8 | 54.8 | 49.7 | 76.4 | 94.8 | 12.4 | 54.1 | 4 |
| Dairy7 - Frt14 - MFE7 - Veg21 | 45.7 | 86.1 | 43.8 | 70.7 | 33.3 | 49 | 42.4 | 179.6 | 71.5 | 11.5 | 52.1 | 4 |
| Dairy7 - Frt14 - MFE7 - SPot7 | 51.7 | 90.2 | 44.4 | 77.4 | 34.4 | 56.9 | 44.4 | 180 | 95.5 | 11.9 | 51.9 | 4 |
| Dairy7 - Frt14 - Veg21 - SPot7 | 50.7 | 91.5 | 44.3 | 73.3 | 31.4 | 54.5 | 44.7 | 76.5 | 95 | 11.4 | 50.7 | 4 |
| Dairy14 - Leg7 - Frt14 - MFE7 | 58 | 84.7 | 45.6 | 86.8 | 33.7 | 49.1 | 47.9 | 199.6 | 74.1 | 12.3 | 59.5 | 4 |
| Dairy14 - Leg7 - Frt14 - Veg21 | 57.1 | 86 | 45.5 | 82.6 | 30.6 | 46.7 | 48.1 | 96.1 | 73.6 | 12.3 | 58.1 | 4 |
| Dairy14 - Leg7 - Frt14 - SPot7 | 63 | 90.1 | 46.5 | 89.7 | 31.8 | 54.8 | 50.2 | 96.5 | 97.6 | 12.4 | 58.1 | 4 |
| Dairy14 - Frt14 - MFE7 - Veg21 | 57.4 | 86.1 | 44.7 | 86.5 | 33.3 | 49 | 42.9 | 199.7 | 74.3 | 11.5 | 55.9 | 4 |
| Dairy14 - Frt14 - MFE7 - SPot7 | 63.3 | 90.2 | 45.6 | 93.6 | 34.4 | 56.9 | 45 | 200.1 | 98.2 | 11.9 | 55.8 | 4 |
| Dairy14 - Frt14 - Veg21 - SPot7 | 62.4 | 91.5 | 45.2 | 89.2 | 31.4 | 54.5 | 45.2 | 96.6 | 97.8 | 11.4 | 54.4 | 4 |
| Leg7 - Frt14 - MFE7 - WGrt14 | 34.8 | 84.6 | 49.3 | 57.2 | 37.3 | 54.9 | 48.1 | 159.4 | 68.6 | 19.7 | 70.1 | 4 |
| Leg7 - Frt14 - SPot7 - WGrt14 | 39.9 | 90 | 54.8 | 62 | 39.1 | 66.5 | 52.4 | 56.3 | 92.1 | 23.3 | 75 | 4 |
| Frt14 - MFE7 - Veg21 - WGrt14 | 34.1 | 86 | 47.6 | 56.7 | 36.3 | 53.5 | 42.7 | 159.4 | 68.8 | 18.3 | 66 | 4 |
| Dairy7 - Leg7 - Frt14 - MFE7 - Veg21 | 46.5 | 86.1 | 44.9 | 71.1 | 34.1 | 49.6 | 47.8 | 179.6 | 71.5 | 12.4 | 55.7 | 4 |
| Dairy7 - Leg7 - Frt14 - MFE7 - SPot7 | 52.4 | 90.2 | 45.6 | 78 | 35.2 | 57.9 | 49.8 | 180 | 95.5 | 13 | 55.6 | 4 |
| Dairy7 - Leg7 - Frt14 - Veg21 - SPot7 | 51.4 | 91.5 | 45.4 | 73.7 | 32.2 | 55.3 | 50.1 | 76.5 | 95 | 12.5 | 54.2 | 4 |
| Dairy7 - Frt14 - MFE7 - Veg21 - SPot7 | 51.8 | 91.6 | 44.6 | 77.6 | 34.9 | 57.4 | 44.8 | 180 | 95.7 | 12 | 52.1 | 4 |
| Dairy14 - Leg7 - Frt14 - MFE7 - Veg21 | 58.1 | 86.1 | 45.8 | 86.9 | 34.1 | 49.6 | 48.3 | 199.7 | 74.3 | 12.4 | 59.6 | 4 |
| Dairy14 - Leg7 - Frt14 - MFE7 - SPot7 | 64 | 90.2 | 47.3 | 94.3 | 35.2 | 57.9 | 50.4 | 200.1 | 98.2 | 13 | 60 | 4 |
| Dairy14 - Leg7 - Frt14 - Veg21 - SPot7 | 63.1 | 91.5 | 46.7 | 89.9 | 32.2 | 55.3 | 50.6 | 96.6 | 97.8 | 12.5 | 58.3 | 4 |
| Dairy14 - Frt14 - MFE7 - Veg21 - SPot7 | 63.4 | 91.6 | 45.8 | 93.7 | 34.9 | 57.4 | 45.3 | 200.2 | 98.4 | 12 | 56 | 4 |
| Leg7 - Frt14 - MFE7 - Veg21 - WGrt14 | 34.9 | 86 | 49.5 | 57.4 | 37.7 | 55.5 | 48.5 | 159.4 | 68.8 | 19.8 | 70.3 | 4 |
| Leg7 - Frt14 - Veg21 - SPot7 - WGrt14 | 40 | 91.4 | 55 | 62.2 | 39.5 | 67.1 | 52.7 | 56.4 | 92.3 | 23.5 | 75.3 | 4 |
| Dairy7 - Leg7 - Frt14 - MFE7 - Veg21 - SPot7 | 52.5 | 91.6 | 45.7 | 78.2 | 35.6 | 58.4 | 50.2 | 180 | 95.7 | 13.1 | 55.7 | 4 |
| Dairy14 - Leg7 - Frt14 - MFE7 - Veg21 - SPot7 | 64.1 | 91.6 | 47.5 | 94.5 | 35.6 | 58.4 | 50.7 | 200.2 | 98.4 | 13.1 | 60.2 | 4 |
| Dairy14 - Leg14 | 56.2 | 71 | 46.4 | 78.4 | 29.5 | 33.2 | 55.5 | 95.6 | 71.7 | 13.3 | 60 | 4 |
| Dairy7 - Leg14 - MFE7 | 45.6 | 71.1 | 45.8 | 66.9 | 32.9 | 36.3 | 55.1 | 179.1 | 69.7 | 13.4 | 57.7 | 4 |
| Dairy7 - Leg14 - SPot7 | 50.6 | 76.5 | 46.3 | 69.5 | 31.1 | 44.1 | 57.4 | 75.9 | 93.1 | 14.9 | 56.3 | 4 |
| Dairy7 - Leg14 - WGrt14 | 44.6 | 70.9 | 51.3 | 64.9 | 32.5 | 44.9 | 56.5 | 75.5 | 69 | 21.1 | 78 | 4 |
| Dairy14 - Leg14 - Frt7 | 56.9 | 76.6 | 46.4 | 79.5 | 30 | 38.2 | 57.7 | 95.8 | 72.3 | 14.1 | 60.5 | 4 |
| Dairy14 - Leg14 - MFE7 | 57.2 | 71.1 | 46.6 | 82.7 | 32.9 | 36.3 | 55.6 | 199.2 | 72.4 | 13.4 | 61.6 | 4 |
| Dairy14 - Leg14 - Veg21 | 56.3 | 72.4 | 46.5 | 78.5 | 29.9 | 33.6 | 55.9 | 95.7 | 71.9 | 13.3 | 60.2 | 4 |
| Dairy14 - Leg14 - SPot7 | 62.2 | 76.5 | 47.4 | 85.6 | 31.1 | 44.8 | 58 | 96.1 | 95.9 | 14.9 | 60.9 | 4 |
| Leg14 - MFE7 - WGrt14 | 34 | 71 | 50 | 53.1 | 36.3 | 47.5 | 55.8 | 158.9 | 66.9 | 22.1 | 74.7 | 4 |
| Dairy7 - Leg14 - Frt7 - MFE7 | 46.3 | 76.7 | 45.8 | 68 | 33.4 | 41.1 | 57.3 | 179.3 | 70.3 | 14.2 | 58.2 | 4 |
| Dairy7 - Leg14 - Frt7 - SPot7 | 51.3 | 82.1 | 46.3 | 70.8 | 31.6 | 48.4 | 59.6 | 76.2 | 93.7 | 15.1 | 56.7 | 4 |
| Dairy7 - Leg14 - MFE7 - Veg21 | 45.7 | 72.5 | 45.9 | 67 | 33.3 | 36.8 | 55.5 | 179.1 | 69.9 | 13.5 | 57.8 | 4 |
| Dairy7 - Leg14 - MFE7 - SPot7 | 51.6 | 76.6 | 46.6 | 73.9 | 34.5 | 47.8 | 57.6 | 179.5 | 93.8 | 15.4 | 58.2 | 4 |
| Dairy7 - Leg14 - Veg21 - SPot7 | 50.7 | 77.9 | 46.5 | 69.6 | 31.5 | 44.6 | 57.8 | 76 | 93.3 | 15 | 56.5 | 4 |
| Dairy14 - Leg14 - Frt7 - MFE7 | 57.9 | 76.7 | 46.7 | 83.9 | 33.4 | 41.1 | 57.8 | 199.4 | 73 | 14.2 | 62 | 4 |
| Dairy14 - Leg14 - Frt7 - Veg21 | 57 | 78 | 46.6 | 79.7 | 30.4 | 38.7 | 58 | 95.9 | 72.5 | 14.1 | 60.7 | 4 |
| Dairy14 - Leg14 - Frt7 - SPot7 | 62.9 | 82.1 | 48 | 87 | 31.6 | 48.5 | 60.1 | 96.3 | 96.5 | 15.1 | 61.4 | 4 |
| Dairy14 - Leg14 - MFE7 - Veg21 | 57.3 | 72.5 | 46.8 | 82.9 | 33.3 | 36.8 | 56 | 199.2 | 72.6 | 13.5 | 61.7 | 4 |
| Dairy14 - Leg14 - MFE7 - SPot7 | 63.3 | 76.6 | 48.2 | 90.1 | 34.5 | 48.7 | 58.3 | 199.7 | 96.5 | 15.4 | 63 | 4 |
| Dairy14 - Leg14 - Veg21 - SPot7 | 62.3 | 77.9 | 47.5 | 85.8 | 31.5 | 45.3 | 58.4 | 96.1 | 96.1 | 15 | 61.1 | 4 |
| Leg14 - Frt7 - MFE7 - WGrt14 | 34.7 | 76.7 | 51.1 | 54.6 | 37.5 | 50.8 | 58 | 159.1 | 67.5 | 22.5 | 74.8 | 4 |
| Leg14 - MFE7 - Veg21 - WGrt14 | 34.1 | 72.5 | 50.2 | 53.3 | 36.7 | 48.1 | 56.2 | 159 | 67.1 | 22.2 | 74.9 | 4 |
| Leg14 - MFE7 - SPot7 - WGrt14 | 40.2 | 76.6 | 56.6 | 62.6 | 41.9 | 64.6 | 60.5 | 159.4 | 91.1 | 26.7 | 82.8 | 4 |
| Dairy7 - Leg14 - Frt7 - MFE7 - Veg21 | 46.4 | 78.1 | 45.9 | 68.2 | 33.8 | 41.6 | 57.7 | 179.3 | 70.5 | 14.2 | 58.3 | 4 |
| Dairy7 - Leg14 - Frt7 - MFE7 - SPot7 | 52.3 | 82.2 | 46.9 | 75.3 | 35 | 51.7 | 59.7 | 179.7 | 94.4 | 15.6 | 58.7 | 4 |
| Dairy7 - Leg14 - Frt7 - Veg21 - SPot7 | 51.4 | 83.5 | 46.5 | 70.9 | 32 | 48.9 | 60 | 76.2 | 93.9 | 15.2 | 56.9 | 4 |
| Dairy7 - Leg14 - MFE7 - Veg21 - SPot7 | 51.7 | 78 | 46.7 | 74.1 | 34.9 | 48.3 | 57.9 | 179.6 | 94 | 15.6 | 58.4 | 4 |
| Dairy14 - Leg14 - Frt7 - MFE7 - Veg21 | 58 | 78.1 | 46.8 | 84 | 33.8 | 41.6 | 58.2 | 199.4 | 73.2 | 14.2 | 62.2 | 4 |
| Dairy14 - Leg14 - Frt7 - MFE7 - SPot7 | 64 | 82.2 | 49.1 | 91.6 | 35 | 52.2 | 60.3 | 199.9 | 97.1 | 15.6 | 63.4 | 4 |
| Dairy14 - Leg14 - Frt7 - Veg21 - SPot7 | 63 | 83.5 | 48.2 | 87.1 | 32 | 49 | 60.5 | 96.3 | 96.7 | 15.2 | 61.6 | 4 |
| Dairy14 - Leg14 - MFE7 - Veg21 - SPot7 | 63.4 | 78 | 48.4 | 90.3 | 34.9 | 49.2 | 58.7 | 199.7 | 96.7 | 15.6 | 63.3 | 4 |
| Leg14 - Frt7 - MFE7 - Veg21 - WGrt14 | 34.8 | 78.1 | 51.3 | 54.8 | 37.9 | 51.4 | 58.4 | 159.2 | 67.7 | 22.6 | 75 | 4 |
| Dairy7 - Leg14 - Frt7 - MFE7 - Veg21 - SPot7 | 52.4 | 83.6 | 47.1 | 75.4 | 35.4 | 52.3 | 60.1 | 179.8 | 94.6 | 15.8 | 58.9 | 4 |
| Dairy14 - Leg14 - Frt7 - MFE7 - Veg21 - SPot7 | 64.1 | 83.6 | 49.3 | 91.8 | 35.4 | 52.7 | 60.6 | 199.9 | 97.3 | 15.8 | 63.6 | 4 |
| Dairy7 - Leg14 - Frt14 | 47.4 | 85.5 | 47.4 | 68.4 | 32.6 | 49.3 | 63.3 | 75.9 | 70.7 | 17.6 | 60.6 | 4 |
| Dairy14 - Leg14 - Frt14 | 59 | 85.5 | 48.3 | 84.2 | 32.6 | 49.3 | 63.8 | 96.1 | 73.4 | 17.6 | 64.4 | 4 |
| Dairy7 - Leg14 - Frt14 - MFE7 | 48.4 | 85.6 | 47.7 | 72.7 | 36 | 52.2 | 63.4 | 179.5 | 71.3 | 17.7 | 62.1 | 4 |
| Dairy7 - Leg14 - Frt14 - Veg21 | 47.5 | 86.9 | 47.6 | 68.5 | 33 | 49.8 | 63.7 | 76 | 70.9 | 17.7 | 60.7 | 4 |
| Dairy14 - Leg14 - Frt14 - Veg21 | 59.1 | 86.9 | 48.5 | 84.4 | 33 | 49.8 | 64.2 | 96.1 | 73.6 | 17.7 | 64.6 | 4 |
| Leg14 - Frt14 - MFE7 - SPot7 | 42.8 | 91.1 | 47.6 | 63.9 | 37.6 | 61.7 | 65.3 | 159.9 | 92.8 | 19 | 58.2 | 4 |
| Leg14 - Frt14 - MFE7 - WGrt14 | 36.9 | 85.6 | 53.3 | 59.5 | 40.4 | 59.8 | 64.7 | 159.4 | 68.6 | 25.9 | 78 | 4 |
| Dairy7 - Leg14 - Frt14 - MFE7 - Veg21 | 48.5 | 87 | 47.8 | 72.9 | 36.4 | 52.7 | 63.8 | 179.6 | 71.5 | 17.8 | 62.2 | 4 |
| Leg14 - Frt14 - MFE7 - Veg21 - SPot7 | 42.9 | 92.5 | 47.7 | 64.1 | 38 | 62.2 | 65.7 | 159.9 | 93 | 19.1 | 58.3 | 4 |
| Dairy7 | 42 | 70.4 | 42.9 | 61.2 | 28.1 | 31.1 | 37.4 | 75.5 | 69 | 9.7 | 47.1 | 3 |
| MFE7 | 31.4 | 70.5 | 42.3 | 49.7 | 31.5 | 34 | 37.1 | 158.9 | 66.9 | 9.8 | 44.7 | 3 |
| Dairy7 - Leg7 | 42.7 | 70.4 | 43.4 | 61.4 | 28.3 | 31.7 | 42.5 | 75.5 | 69 | 10.2 | 50.5 | 3 |
| Dairy7 - Frt7 | 42.7 | 76 | 42.9 | 62.3 | 28.6 | 36 | 39.2 | 75.7 | 69.6 | 9.8 | 47.4 | 3 |
| Dairy7 - Veg21 | 42.1 | 71.8 | 43.1 | 61.4 | 28.5 | 31.6 | 37.8 | 75.5 | 69.2 | 9.8 | 47.2 | 3 |
| Leg7 - MFE7 | 32.1 | 70.5 | 42.8 | 49.9 | 31.7 | 34.8 | 42.2 | 158.9 | 66.9 | 10.3 | 48.1 | 3 |
| Leg7 - WGrt14 | 31.1 | 70.4 | 45.3 | 46.9 | 30.2 | 40.8 | 42.8 | 55.3 | 66.3 | 17.3 | 65.4 | 3 |
| Frt7 - MFE7 | 32.1 | 76.1 | 42.3 | 50.8 | 32 | 38.9 | 38.8 | 159.1 | 67.5 | 9.9 | 45.1 | 3 |
| MFE7 - Veg21 | 31.5 | 71.9 | 42.4 | 49.9 | 31.9 | 34.5 | 37.4 | 159 | 67.1 | 9.9 | 44.9 | 3 |
| MFE7 - SPot7 | 37.4 | 76 | 43.1 | 56.6 | 33 | 44.2 | 39.5 | 159.4 | 91.1 | 11 | 44.7 | 3 |
| MFE7 - WGrt14 | 31.4 | 70.5 | 45.1 | 51 | 33.2 | 42.8 | 37.8 | 158.9 | 66.9 | 16.9 | 63.7 | 3 |
| SPot7 - WGrt14 | 36.5 | 75.9 | 49.3 | 54.9 | 33.8 | 54.5 | 41.1 | 55.8 | 90.4 | 20.1 | 67.3 | 3 |
| Dairy7 - Leg7 - Frt7 | 43.4 | 76 | 43.4 | 62.5 | 28.8 | 36.6 | 44.5 | 75.7 | 69.6 | 10.3 | 50.8 | 3 |
| Dairy7 - Leg7 - Veg21 | 42.8 | 71.8 | 43.6 | 61.6 | 28.7 | 32.2 | 42.9 | 75.5 | 69.2 | 10.3 | 50.6 | 3 |
| Dairy7 - Frt7 - Veg21 | 42.8 | 77.4 | 43.1 | 62.5 | 29 | 36.5 | 39.6 | 75.7 | 69.8 | 9.8 | 47.5 | 3 |
| Leg7 - Frt7 - MFE7 | 32.8 | 76.1 | 42.8 | 51 | 32.2 | 39.4 | 44.1 | 159.1 | 67.5 | 10.4 | 48.4 | 3 |
| Leg7 - Frt7 - WGrt14 | 31.8 | 76 | 46.3 | 48.4 | 31.4 | 44.3 | 44.8 | 55.5 | 66.9 | 17.5 | 65.4 | 3 |
| Leg7 - MFE7 - Veg21 | 32.2 | 71.9 | 42.9 | 50.1 | 32.1 | 35.3 | 42.5 | 159 | 67.1 | 10.4 | 48.2 | 3 |
| Leg7 - MFE7 - SPot7 | 38.1 | 76 | 43.6 | 56.8 | 33.3 | 45.5 | 44.6 | 159.4 | 91.1 | 12 | 48.1 | 3 |
| Leg7 - Veg21 - WGrt14 | 31.2 | 71.8 | 45.5 | 47.1 | 30.7 | 41.4 | 43.2 | 55.4 | 66.5 | 17.4 | 65.6 | 3 |
| Leg7 - SPot7 - WGrt14 | 37.2 | 75.9 | 50.8 | 55.7 | 34.9 | 56.8 | 46.8 | 55.8 | 90.4 | 21.6 | 72 | 3 |
| Frt7 - MFE7 - Veg21 | 32.2 | 77.5 | 42.4 | 51 | 32.4 | 39.4 | 39.2 | 159.2 | 67.7 | 9.9 | 45.2 | 3 |
| Frt7 - MFE7 - SPot7 | 38.1 | 81.6 | 43.1 | 57.7 | 33.5 | 48.4 | 41.3 | 159.6 | 91.7 | 11 | 45.1 | 3 |
| Frt7 - MFE7 - WGrt14 | 32.1 | 76.1 | 46.1 | 52.5 | 34.3 | 46.2 | 39.7 | 159.1 | 67.5 | 17 | 63.8 | 3 |
| Frt7 - SPot7 - WGrt14 | 37.2 | 81.5 | 50.4 | 56.5 | 35.1 | 57.4 | 43.3 | 56 | 91 | 20.5 | 67.6 | 3 |
| MFE7 - Veg21 - SPot7 | 37.5 | 77.4 | 43.2 | 56.8 | 33.4 | 44.7 | 39.9 | 159.5 | 91.3 | 11.1 | 44.9 | 3 |
| MFE7 - Veg21 - WGrt14 | 31.5 | 71.9 | 45.3 | 51.2 | 33.6 | 43.4 | 38.1 | 159 | 67.1 | 17 | 64 | 3 |
| Veg21 - SPot7 - WGrt14 | 36.6 | 77.3 | 49.5 | 55.1 | 34.2 | 55.1 | 41.5 | 55.9 | 90.6 | 20.3 | 67.6 | 3 |
| Dairy7 - Leg7 - Frt7 - Veg21 | 43.5 | 77.4 | 43.6 | 62.7 | 29.2 | 37 | 44.9 | 75.7 | 69.8 | 10.4 | 50.9 | 3 |
| Leg7 - Frt7 - MFE7 - Veg21 | 32.9 | 77.5 | 42.9 | 51.2 | 32.6 | 39.9 | 44.5 | 159.2 | 67.7 | 10.5 | 48.6 | 3 |
| Leg7 - Frt7 - MFE7 - SPot7 | 38.8 | 81.6 | 43.6 | 57.9 | 33.8 | 49.7 | 46.6 | 159.6 | 91.7 | 12 | 48.4 | 3 |
| Leg7 - Frt7 - Veg21 - WGrt14 | 31.9 | 77.4 | 46.5 | 48.6 | 31.9 | 44.8 | 45.2 | 55.6 | 67.1 | 17.6 | 65.6 | 3 |
| Leg7 - Frt7 - SPot7 - WGrt14 | 38 | 81.5 | 52.4 | 57.5 | 36.5 | 59.8 | 49.2 | 56 | 91 | 22 | 72.6 | 3 |
| Leg7 - MFE7 - Veg21 - SPot7 | 38.3 | 77.4 | 43.7 | 57 | 33.7 | 46.1 | 45 | 159.5 | 91.3 | 12.1 | 48.3 | 3 |
| Leg7 - Veg21 - SPot7 - WGrt14 | 37.3 | 77.3 | 51 | 55.9 | 35.3 | 57.4 | 47.2 | 55.9 | 90.6 | 21.8 | 72.3 | 3 |
| Frt7 - MFE7 - Veg21 - SPot7 | 38.2 | 83 | 43.2 | 57.9 | 33.9 | 48.9 | 41.6 | 159.7 | 91.9 | 11.2 | 45.2 | 3 |
| Frt7 - MFE7 - Veg21 - WGrt14 | 32.2 | 77.5 | 46.3 | 52.6 | 34.8 | 46.7 | 40.1 | 159.2 | 67.7 | 17.2 | 64 | 3 |
| Frt7 - Veg21 - SPot7 - WGrt14 | 37.3 | 82.9 | 50.7 | 56.7 | 35.6 | 58 | 43.7 | 56.1 | 91.2 | 20.7 | 67.9 | 3 |
| Leg7 - Frt7 - MFE7 - Veg21 - SPot7 | 39 | 83 | 43.7 | 58.1 | 34.2 | 50.2 | 46.9 | 159.7 | 91.9 | 12.1 | 48.6 | 3 |
| Leg7 - Frt7 - Veg21 - SPot7 - WGrt14 | 38.1 | 82.9 | 52.6 | 57.7 | 37 | 60.4 | 49.5 | 56.1 | 91.2 | 22.2 | 72.9 | 3 |
| Frt14 - MFE7 | 34 | 84.6 | 42.7 | 54.7 | 32.9 | 48.6 | 41.5 | 159.4 | 68.6 | 11.4 | 48.2 | 3 |
| Leg7 - Frt14 - MFE7 | 34.7 | 84.6 | 43.9 | 55.1 | 33.7 | 49.1 | 46.9 | 159.4 | 68.6 | 12.3 | 51.8 | 3 |
| Leg7 - Frt14 - WGrt14 | 33.7 | 84.5 | 48.2 | 52.6 | 33.5 | 51.1 | 47.7 | 55.8 | 67.9 | 18.7 | 68 | 3 |
| Frt14 - MFE7 - Veg21 | 34.1 | 86 | 42.9 | 54.8 | 33.3 | 49 | 41.8 | 159.4 | 68.8 | 11.5 | 48.3 | 3 |
| Frt14 - MFE7 - SPot7 | 40 | 90.1 | 43.5 | 61.6 | 34.4 | 56.9 | 43.9 | 159.9 | 92.8 | 11.9 | 48.2 | 3 |
| Frt14 - SPot7 - WGrt14 | 39.1 | 90 | 52.4 | 61 | 37 | 63.6 | 46.3 | 56.3 | 92.1 | 21.8 | 70 | 3 |
| Leg7 - Frt14 - MFE7 - Veg21 | 34.8 | 86 | 44 | 55.2 | 34.1 | 49.6 | 47.2 | 159.4 | 68.8 | 12.4 | 51.9 | 3 |
| Leg7 - Frt14 - MFE7 - SPot7 | 40.7 | 90.1 | 44.7 | 62 | 35.2 | 57.9 | 49.3 | 159.9 | 92.8 | 13 | 51.8 | 3 |
| Leg7 - Frt14 - Veg21 - WGrt14 | 33.8 | 85.9 | 48.4 | 52.8 | 33.9 | 51.6 | 48 | 55.9 | 68.2 | 18.8 | 68.2 | 3 |
| Frt14 - MFE7 - Veg21 - SPot7 | 40.1 | 91.6 | 43.7 | 61.7 | 34.9 | 57.4 | 44.3 | 159.9 | 93 | 12 | 48.3 | 3 |
| Frt14 - Veg21 - SPot7 - WGrt14 | 39.3 | 91.4 | 52.6 | 61.2 | 37.5 | 64.1 | 46.7 | 56.4 | 92.3 | 22 | 70.3 | 3 |
| Leg7 - Frt14 - MFE7 - Veg21 - SPot7 | 40.8 | 91.6 | 44.8 | 62.2 | 35.6 | 58.4 | 49.7 | 159.9 | 93 | 13.1 | 51.9 | 3 |
| Dairy7 - Leg14 | 44.5 | 70.9 | 45.5 | 62.5 | 29.5 | 33.2 | 55 | 75.5 | 69 | 13.3 | 56.2 | 3 |
| Leg14 - MFE7 | 33.9 | 71 | 44.9 | 51 | 32.9 | 36.3 | 54.6 | 158.9 | 66.9 | 13.4 | 53.8 | 3 |
| Leg14 - WGrt14 | 32.9 | 70.9 | 48.9 | 48.5 | 32.5 | 43.4 | 55.5 | 55.3 | 66.3 | 21.1 | 72 | 3 |
| Dairy7 - Leg14 - Frt7 | 45.2 | 76.6 | 45.5 | 63.7 | 30 | 38.2 | 57.2 | 75.7 | 69.6 | 14.1 | 56.6 | 3 |
| Dairy7 - Leg14 - Veg21 | 44.7 | 72.4 | 45.7 | 62.7 | 29.9 | 33.6 | 55.3 | 75.5 | 69.2 | 13.3 | 56.3 | 3 |
| Leg14 - Frt7 - MFE7 | 34.6 | 76.7 | 44.9 | 52.2 | 33.4 | 41.1 | 56.8 | 159.1 | 67.5 | 14.2 | 54.3 | 3 |
| Leg14 - Frt7 - WGrt14 | 33.6 | 76.6 | 50 | 49.9 | 33.7 | 46.8 | 57.6 | 55.6 | 66.9 | 21.5 | 72 | 3 |
| Leg14 - MFE7 - Veg21 | 34.1 | 72.5 | 45 | 51.2 | 33.3 | 36.8 | 54.9 | 159 | 67.1 | 13.5 | 53.9 | 3 |
| Leg14 - MFE7 - SPot7 | 40 | 76.6 | 45.7 | 57.9 | 34.5 | 47.3 | 57 | 159.4 | 91.1 | 15.4 | 53.8 | 3 |
| Leg14 - Veg21 - WGrt14 | 33 | 72.3 | 49.2 | 48.6 | 32.9 | 43.9 | 55.9 | 55.4 | 66.5 | 21.3 | 72.2 | 3 |
| Leg14 - SPot7 - WGrt14 | 39.1 | 76.4 | 55.1 | 57.6 | 37.8 | 60 | 59.9 | 55.8 | 90.4 | 25.7 | 79.6 | 3 |
| Dairy7 - Leg14 - Frt7 - Veg21 | 45.3 | 78 | 45.7 | 63.8 | 30.4 | 38.7 | 57.5 | 75.7 | 69.8 | 14.1 | 56.8 | 3 |
| Leg14 - Frt7 - MFE7 - Veg21 | 34.7 | 78.1 | 45 | 52.3 | 33.8 | 41.6 | 57.1 | 159.2 | 67.7 | 14.2 | 54.4 | 3 |
| Leg14 - Frt7 - MFE7 - SPot7 | 40.7 | 82.2 | 45.7 | 59.1 | 35 | 51.7 | 59.2 | 159.6 | 91.7 | 15.6 | 54.3 | 3 |
| Leg14 - Frt7 - Veg21 - WGrt14 | 33.8 | 78 | 50.2 | 50.1 | 34.1 | 47.4 | 57.9 | 55.6 | 67.1 | 21.6 | 72.3 | 3 |
| Leg14 - Frt7 - SPot7 - WGrt14 | 39.9 | 82.1 | 56.7 | 59.5 | 39.5 | 63 | 62.3 | 56.1 | 91 | 26.1 | 80.2 | 3 |
| Leg14 - MFE7 - Veg21 - SPot7 | 40.1 | 78 | 45.8 | 58.1 | 34.9 | 47.8 | 57.4 | 159.5 | 91.3 | 15.6 | 54 | 3 |
| Leg14 - Veg21 - SPot7 - WGrt14 | 39.2 | 77.8 | 55.3 | 57.8 | 38.2 | 60.6 | 60.2 | 55.9 | 90.6 | 25.8 | 79.9 | 3 |
| Leg14 - Frt7 - MFE7 - Veg21 - SPot7 | 40.8 | 83.6 | 45.8 | 59.3 | 35.4 | 52.2 | 59.6 | 159.7 | 91.9 | 15.8 | 54.4 | 3 |
| Leg14 - Frt7 - Veg21 - SPot7 - WGrt14 | 40 | 83.5 | 57 | 59.7 | 39.9 | 63.6 | 62.6 | 56.1 | 91.2 | 26.2 | 80.5 | 3 |
| Leg14 - Frt14 - MFE7 | 36.8 | 85.6 | 46.8 | 56.9 | 36 | 52.2 | 62.9 | 159.4 | 68.6 | 17.7 | 58.2 | 3 |
| Leg14 - Frt14 - SPot7 | 41.8 | 91 | 47.3 | 59.5 | 34.2 | 58.4 | 65.2 | 56.3 | 92.1 | 18.4 | 56.7 | 3 |
| Leg14 - Frt14 - WGrt14 | 35.8 | 85.5 | 52.1 | 54.8 | 36.5 | 55.9 | 64.2 | 55.8 | 67.9 | 24.9 | 75.4 | 3 |
| Leg14 - Frt14 - MFE7 - Veg21 | 36.9 | 87 | 47 | 57 | 36.4 | 52.7 | 63.3 | 159.4 | 68.8 | 17.8 | 58.3 | 3 |
| Leg14 - Frt14 - Veg21 - SPot7 | 41.9 | 92.4 | 47.5 | 59.6 | 34.6 | 58.9 | 65.6 | 56.3 | 92.3 | 18.5 | 56.9 | 3 |
| Leg14 - Frt14 - Veg21 - WGrt14 | 35.9 | 86.9 | 52.3 | 54.9 | 36.9 | 56.4 | 64.6 | 55.9 | 68.2 | 25.1 | 75.7 | 3 |
| Leg7 | 31.1 | 70.4 | 42.5 | 45.6 | 28.3 | 31.7 | 42 | 55.3 | 66.3 | 10.2 | 46.6 | 2 |
| Leg14 | 32.9 | 70.9 | 44.6 | 46.7 | 29.5 | 33.2 | 54.5 | 55.3 | 66.3 | 13.3 | 52.3 | 2 |
| Frt7 | 31 | 76 | 42 | 46.5 | 28.6 | 36 | 38.7 | 55.5 | 66.9 | 9.8 | 43.6 | 2 |
| Frt14 | 32.9 | 84.5 | 42.4 | 50.3 | 29.5 | 45.7 | 41.4 | 55.8 | 67.9 | 11.3 | 46.7 | 2 |
| Veg21 | 30.5 | 71.8 | 42.2 | 45.5 | 28.5 | 31.6 | 37.3 | 55.4 | 66.5 | 9.8 | 43.4 | 2 |
| GLV2 | 33.8 | 85.3 | 42.8 | 47.9 | 29 | 34.7 | 39.4 | 55.4 | 74.6 | 11.2 | 45.2 | 2 |
| SPot7 | 36.4 | 75.9 | 42.8 | 52.3 | 29.6 | 40.8 | 39.4 | 55.8 | 90.4 | 10.4 | 43.3 | 2 |
| WGrt14 | 30.4 | 70.4 | 44 | 46.4 | 29.4 | 38.9 | 37.4 | 55.3 | 66.3 | 16.2 | 61.4 | 2 |
| Leg7 - Frt7 | 31.8 | 76 | 42.5 | 46.7 | 28.8 | 36.6 | 44 | 55.5 | 66.9 | 10.3 | 46.9 | 2 |
| Leg7 - Veg21 | 31.2 | 71.8 | 42.7 | 45.7 | 28.7 | 32.2 | 42.4 | 55.4 | 66.5 | 10.3 | 46.8 | 2 |
| Leg7 - SPot7 | 37.1 | 75.9 | 43.3 | 52.5 | 29.8 | 42.2 | 44.5 | 55.8 | 90.4 | 11.4 | 46.6 | 2 |
| Frt7 - Veg21 | 31.2 | 77.4 | 42.2 | 46.6 | 29 | 36.5 | 39.1 | 55.6 | 67.1 | 9.8 | 43.8 | 2 |
| Frt7 - SPot7 | 37.1 | 81.5 | 42.8 | 53.4 | 30.1 | 45 | 41.1 | 56 | 91 | 10.5 | 43.6 | 2 |
| Frt7 - WGrt14 | 31 | 76 | 45 | 47.8 | 30.5 | 42.3 | 39.4 | 55.5 | 66.9 | 16.3 | 61.4 | 2 |
| Veg21 - SPot7 | 36.5 | 77.3 | 43 | 52.5 | 30 | 41.3 | 39.7 | 55.9 | 90.6 | 10.5 | 43.4 | 2 |
| Veg21 - WGrt14 | 30.5 | 71.8 | 44.2 | 46.6 | 29.8 | 39.5 | 37.8 | 55.4 | 66.5 | 16.3 | 61.6 | 2 |
| Leg7 - Frt7 - Veg21 | 31.9 | 77.4 | 42.7 | 46.8 | 29.2 | 37 | 44.3 | 55.6 | 67.1 | 10.4 | 47.1 | 2 |
| Leg7 - Frt7 - SPot7 | 37.8 | 81.5 | 43.3 | 53.6 | 30.3 | 46.3 | 46.4 | 56 | 91 | 11.4 | 46.9 | 2 |
| Leg7 - Veg21 - SPot7 | 37.2 | 77.3 | 43.5 | 52.7 | 30.2 | 42.7 | 44.8 | 55.9 | 90.6 | 11.5 | 46.8 | 2 |
| Frt7 - Veg21 - SPot7 | 37.2 | 82.9 | 43 | 53.6 | 30.5 | 45.5 | 41.5 | 56.1 | 91.2 | 10.6 | 43.8 | 2 |
| Frt7 - Veg21 - WGrt14 | 31.2 | 77.4 | 45.2 | 48 | 31 | 42.8 | 39.8 | 55.6 | 67.1 | 16.4 | 61.6 | 2 |
| Leg7 - Frt7 - Veg21 - SPot7 | 37.9 | 82.9 | 43.5 | 53.8 | 30.7 | 46.9 | 46.8 | 56.1 | 91.2 | 11.5 | 47.1 | 2 |
| Leg7 - Frt14 | 33.6 | 84.5 | 43.6 | 50.8 | 30.2 | 46.2 | 46.7 | 55.8 | 67.9 | 12.2 | 50.3 | 2 |
| Frt14 - Veg21 | 33 | 85.9 | 42.6 | 50.5 | 29.9 | 46.2 | 41.7 | 55.9 | 68.2 | 11.3 | 46.9 | 2 |
| Frt14 - SPot7 | 39 | 90 | 43.2 | 57.3 | 31 | 54 | 43.8 | 56.3 | 92.1 | 11.4 | 46.7 | 2 |
| Frt14 - WGrt14 | 32.9 | 84.5 | 46.3 | 51.9 | 32 | 49 | 42 | 55.8 | 67.9 | 17.3 | 63.7 | 2 |
| Leg7 - Frt14 - Veg21 | 33.8 | 85.9 | 43.8 | 50.9 | 30.6 | 46.7 | 47.1 | 55.9 | 68.2 | 12.3 | 50.5 | 2 |
| Leg7 - Frt14 - SPot7 | 39.7 | 90 | 44.4 | 57.7 | 31.8 | 54.8 | 49.2 | 56.3 | 92.1 | 12.4 | 50.3 | 2 |
| Frt14 - Veg21 - SPot7 | 39.1 | 91.4 | 43.4 | 57.4 | 31.4 | 54.5 | 44.2 | 56.3 | 92.3 | 11.4 | 46.9 | 2 |
| Frt14 - Veg21 - WGrt14 | 33.1 | 85.9 | 46.6 | 52 | 32.4 | 49.6 | 42.4 | 55.9 | 68.2 | 17.4 | 63.9 | 2 |
| Leg7 - Frt14 - Veg21 - SPot7 | 39.8 | 91.4 | 44.6 | 57.8 | 32.2 | 55.3 | 49.5 | 56.3 | 92.3 | 12.5 | 50.5 | 2 |
| Leg14 - Frt7 | 33.6 | 76.6 | 44.6 | 47.8 | 30 | 38.2 | 56.6 | 55.5 | 66.9 | 14.1 | 52.8 | 2 |
| Leg14 - Veg21 | 33 | 72.3 | 44.8 | 46.8 | 29.9 | 33.6 | 54.8 | 55.4 | 66.5 | 13.3 | 52.4 | 2 |
| Leg14 - SPot7 | 38.9 | 76.4 | 45.4 | 53.6 | 31.1 | 43.9 | 56.9 | 55.8 | 90.4 | 14.9 | 52.3 | 2 |
| Leg14 - Frt7 - Veg21 | 33.7 | 78 | 44.8 | 48 | 30.4 | 38.7 | 57 | 55.6 | 67.1 | 14.1 | 52.9 | 2 |
| Leg14 - Frt7 - SPot7 | 39.6 | 82.1 | 45.4 | 54.8 | 31.6 | 48.4 | 59.1 | 56 | 91 | 15.1 | 52.8 | 2 |
| Leg14 - Veg21 - SPot7 | 39 | 77.8 | 45.6 | 53.8 | 31.5 | 44.4 | 57.3 | 55.9 | 90.6 | 15 | 52.4 | 2 |
| Leg14 - Frt7 - Veg21 - SPot7 | 39.7 | 83.5 | 45.6 | 54.9 | 32 | 48.9 | 59.4 | 56.1 | 91.2 | 15.2 | 52.9 | 2 |
| Leg14 - Frt14 | 35.7 | 85.5 | 46.5 | 52.5 | 32.6 | 49.3 | 62.8 | 55.8 | 67.9 | 17.6 | 56.7 | 2 |
| Leg14 - Frt14 - Veg21 | 35.8 | 86.9 | 46.7 | 52.7 | 33 | 49.8 | 63.1 | 55.9 | 68.2 | 17.7 | 56.9 | 2 |

^‡^ Abbreviations:^:^ Vit - vitamin; Dairy14 – 14 servings of milk per week; Dairy7 – 7 servings of milk per week; Leg14 – 14 servings of legumes per week; Leg7 – 7 servings of legumes per week; Frt14 – 14 servings of fruits per week; Frt7 – 7 servings of fruits per week; Spot7 – 7 servings of sweet potatoes per week; Wgrt14 – 14 servings of whole grain cereals per week; MFE7 – 7 servings of meat, fish or eggs per week; Veg21 – 21 servings of vegetables per week; GLV4 – 4 servings of green leafy vegetables per week

^§^ number of nutrients that were ≥65% in the Module III minimised diet for that nutrients

**Supplementary Material Table 6:** Ordered lists of the three best food sources of nutrients in the nutritionally best diet (Module II), and expressed per kcal, per average portion and per maximum portion per week

| **Calcium** | **Vit C** | **Thiamine** | **Riboflavin** | **Niacin** | **Vitamin B-6** | **Folate** | **Vitamin B-12** | **Vitamin A** | **Iron** | **Zinc** |
| --- | --- | --- | --- | --- | --- | --- | --- | --- | --- | --- |
| Foods that contributed the highest percentage of the nutrient in the Module II, nutritionally best diet | | | | | | | | | | |
| Cows’ milk | Papaya | Millet flour | Cows’ milk | Millet flour | Sweet potato, white | Kidney beans, dry | Mukene | Sweet potato, y | Kidney beans, dry | Millet flour |
| Sweet potato, white | Orange | Sweet potato, white | Millet flour | Mukene | Millet flour | Millet flour | Cows’ milk | Kale | Millet flour | Cows’ milk |
| Sweet potato, y | Cabbage | Cows’ milk | Sweet potato, white | Sweet potato, white | Banana | Kulakula nut flour | Egg | Cows’ milk | Sweet potato, white | Kidney beans, dry |
| Foods with the highest nutrient content per 100 kcal | | | | | | | | | | |
| Amaranth | Kale | Tomato | Amaranth | Tomato | Amaranth | Amaranth | Mukene | Carrot | Amaranth | Amaranth |
| Kale | Papaya | Kulakula nut flour | Pumpkin | Amaranth | Kale | Kulakula nut flour | Egg | Kale | Kale | Sesame paste |
| Cows’ milk | Oranges | Kale | Cows’ milk | Eggplant | Banana | Papaya | Milk | Pumpkin | Sesame paste | Tomato |
| Foods with the highest nutrient content per average serving size | | | | | | | | | | |
| Amaranth | Papaya | Cassava flour | Millet flour | Millet flour | Cassava flour | Kidney beans, dry | Mukene | Kale | Unrefined maize flour, y | Unrefined maize flour, y |
| Cows’ milk | Kale | Millet flour | Cows’ milk | Unrefined maize flour, y | Yams | Kulakula nut flour | Cows’ milk | Sweet potato, y | Millet flour | Millet flour |
| Sesame paste | Cassava flour | Unrefined maize flour, y | Unrefined maize flour, y | Groundnuts, dry | Sweet potato, white | Papaya | Egg | Amaranth | Amaranth | Unrefined maize flour |
| Foods with the highest nutrient content per maximum grams per week that could be included in modelled diets | | | | | | | | | | |
| Cows’ milk | Papaya | Unrefined maize flour | Cows’ milk | Unrefined maize flour | Unrefined maize flour | Kidney beans, dry | Mukene | Sweet potato, y | Unrefined maize flour | Unrefined maize flour |
| Sweet potato, either | Oranges | Millet flour | Unrefined maize flour | Millet flour | Sweet potato, w | Unrefined maize flour | Cows’ milk | Kale | Kidney beans, dry | Millet flour |
| Jack fruit | Cassava flour | Sweet potato, w | Millet flour | Sweet potato, white | Sweet potato, y | Millet flour | Egg | Cows’ milk | Millet flour | Cows’ milk |

**Supplementary material Table 7:** Sensitivity analyses for the variance ratios used to estimate the percentage of the population at risk of inadequate nutrient intakes

| **Nutrient** | **NHANES Variance ratio** | **% at risk with NHANES** | **% at risk if variance ratio = 0.2** | **% at risk if variance ratio = 0.5** | **% at risk if variance ratio = 0.65** | **% at risk if variance ratio = 0.8** |
| --- | --- | --- | --- | --- | --- | --- |
| Protein | 0.297 | 16.8 | 19.1 | 11.4 | 6.6 | 1.9 |
| Calcium | 0.422 | 97.0 | 95.3 | 97.6 | 98.9 | 99.8 |
| Iron | 0.397 | 100 | 100 | 100 | 100 | 100 |
| Zinc | 0.467 | 54.7 | 55.8 | 54.6 | 53.8 | 52.8 |
| Vitamin C | 0.418 | 0 | 0 | 0 | 0 | 0 |
| Vitamin A | 0.515 | 0 | 0 | 0 | 0 | 0 |
| Thiamine | 0.383 | 79.9 | 78.4 | 81.3 | 84.2 | 89.3 |
| Riboflavin | 0.398 | 57.2 | 62.4 | 54.0 | 47.7 | 38.0 |
| Niacin | 0.40 | 91.5 | 89.6 | 92.8 | 95.2 | 98.2 |
| Vitamin B6 | 0.359 | 51.3 | 53.1 | 49.3 | 46.5 | 41.7 |
| Folate | 0.389 | 81.7 | 80.6 | 82.7 | 85.1 | 89.6 |

**References**

FAO/WHO/UNU (2004) Human energy requirements*.* In: Report of a Joint FAO/WHO/UNU Expert Consultation, FAO Food and Nutrition Technical Report Series #1. FAO: Rome.

FAO/WHO Joint Report (2004). Vitamin and Mineral Requirements in Human Nutrition (2nd ed.). World Health Organization and Food and Agricultural Organization.

Hotz, C. & Brown, K. (2004). Assessment of the risk of zinc deficiency in populations and options for its control. *Food & Nutrition Bulletin,* 25(1 suppl 2), S99-S203.
